# Comprehensive analysis method of key risk factors in the subway operation accident by complex network and accident data

**DOI:** 10.1371/journal.pone.0358549

**Published:** 2026-09-18

**Authors:** Shengxiang Ma, Wei Jiang

**Affiliations:** China University of Mining and Technology (Beijing), School of Emergency Management and Safety Engineering, Beijing, China; City University of Hong Kong, HONG KONG

## Abstract

With the continuous expansion of subway systems in China, the operational systems of subway have become increasingly complex, leading to a rise in risk factors. In the event of an accident, these risks can significantly impact the safety and health of individuals, and pose a direct threat to the reliability, social stability, and economic sustainability of subway systems. To effectively prevent the occurrence of operational accidents and ensure the sustainable development of subway systems, it is essential to explore the key risk factors of subway accidents through appropriate technological approaches. This study proposes an integrated approach combining the 24Model, association rule mining, and complex network theory to conduct in-depth mining and analysis of textual data from accident reports, thereby identifying risk factors and exploring the coupling relationships and importance among them. First, risk factors were extracted by analyzing 76 reports using the 24Model. Then, the Apriori algorithm was applied to derive association rules among the risk factors, based on which a risk factor network model was constructed. Finally, the robustness analysis and mutual information theory were employed to validate the model and identify the key risk factors. The results show that unclear safety responsibilities of employees, safety responsibility of managers, insufficient safety oversight of subcontractors, and lack of targeted content in safety training are the four most critical risk factors. The findings of this study provide important safety management decision-making support for the development of more sustainable subway operational systems.

## 1. Introduction

**Fig 1:**
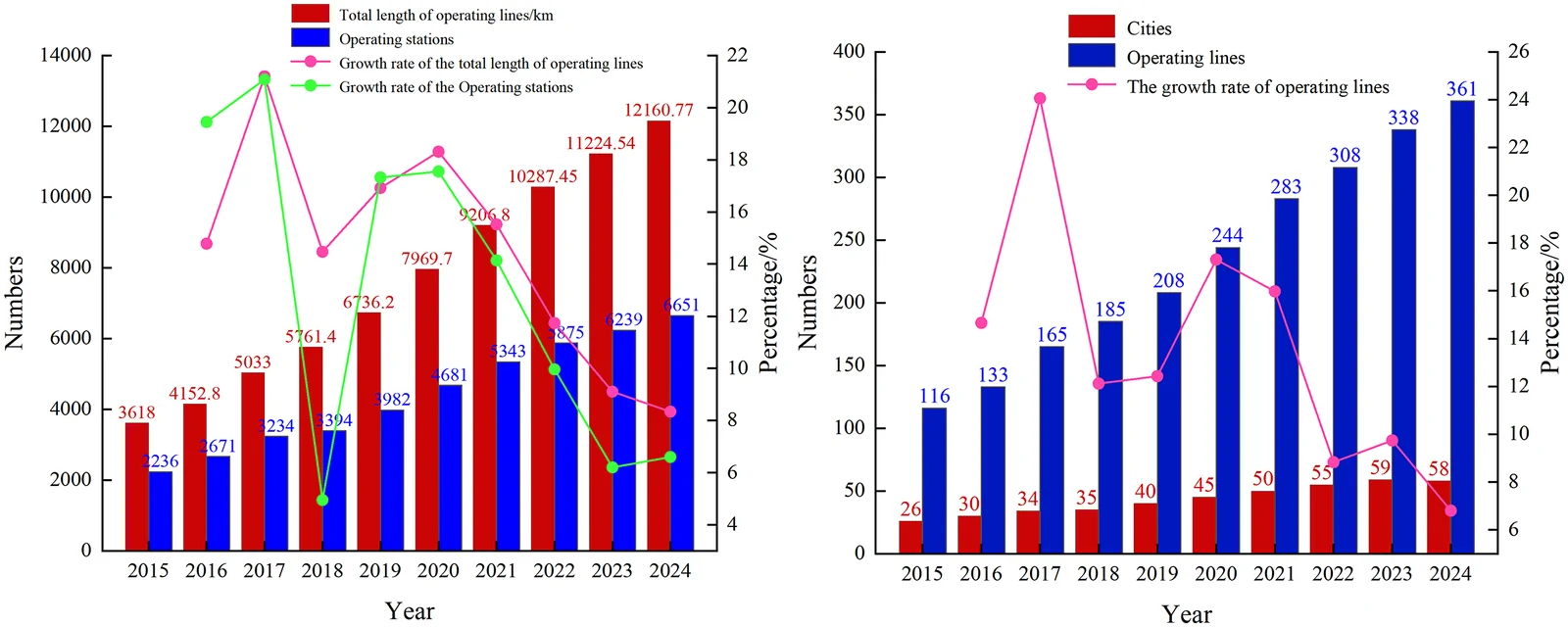
Overview of urban rail transit development in China. The left panel presents the numbers of cities and operating lines and the annual growth rate of operating lines. The right panel presents the total length of operating lines, the number of operating stations, and their corresponding annual growth rates.

In recent years, China’s urban rail transit has experienced rapid and large-scale development, with the length of operational lines ranking first globally for several consecutive years and reaching a world-leading level. By the end of 2024, a total of 361 urban rail transit lines were in operation across 58 cities in mainland China (hereafter referred to as China), covering a cumulative length of 12,160.77 kilometers and serving 6,651 stations, as illustrated in Fig 1. China’s urban rail transit system has entered a period of rapid development; however, safety incidents and operational failures continue to occur frequently during metro operations [1]. Once an operational accident occurs, it may not only disrupt train services but also lead to large-scale passenger congestion, equipment damage, and substantial socioeconomic losses. Therefore, ensuring the safe operation of urban rail transit has become an essential component of the high-quality development of China’s urban rail transit sector.

With the continuous expansion of subway network, the system has become increasingly complex, resulting in heightened uncertainty and vulnerability during operation. Given the numerous subsystems, high passenger mobility, and multifaceted risk factors, the subway system is influenced not only by internal dynamics but also by external disturbances. Consequently, any incident can significantly disrupt system stability and pose serious threats to the safety of urban residents and their property. For instance, on July 20, 2021, a catastrophic rainstorm caused severe flooding in Zhengzhou Metro Line 5, leading to the deaths of 14 passengers [2]; On June 22, 2009, nine people were killed and about 80 injured in a subway crash in Washington [3].

To improve the operational safety of urban rail transit, China has implemented a series of measures, including the continuous improvement of operational safety regulations and technical standards, the establishment of risk classification and control systems together with hazard identification and mitigation mechanisms, and the strengthening of safety training and job responsibility management for operational personnel. Meanwhile, some metro operators have begun to adopt intelligent monitoring technologies to dynamically monitor the operating status of trains, signaling systems, power supply systems, communication systems, and platform facilities. These measures have played a positive role in standardizing safety management practices and preventing equipment failures. However, existing safety management approaches still primarily focus on individual risk control, hazard identification, and post-accident cause investigation, while insufficient attention has been paid to the complex interrelationships and coupling effects among different risk factors. In particular, when numerous accident causative factors interact in a complex manner, conventional analytical methods have difficulty identifying the key risk factors with substantial influence and connectivity from a system-wide perspective. Furthermore, accident investigation reports in China are prepared under a relatively unified management framework, statistical methodology, and accident classification system, resulting in publicly available accident records that provide a relatively complete and consistent data foundation. In contrast, considerable differences exist among countries in terms of accident reporting systems, safety regulatory frameworks, accident classification standards, and data availability. Directly integrating accident data from multiple countries may therefore introduce substantial data heterogeneity, thereby affecting the reliability of risk-factor network construction and subsequent analyses. Therefore, based on subway operation accident data from China and complex network theory, this study constructs a risk-factor association network for subway operation accidents to identify key risk factors from the perspectives of network structure and factor interactions. The findings are expected to provide a more systematic basis for risk prevention and control as well as the allocation of safety management resources.

Current research on subway operation accidents primarily focuses on causation analysis and risk assessment. In terms of causation analysis, The existing research can be categorized into two perspectives: the engineering and technical perspective and the data-driven perspective. The engineering and technical perspective focuses on direct physical causes of accidents. For example, Yao et al. [4] employed the finite element method to investigate the factors influencing passenger injuries in subway collision accidents. In contrast, the data-driven perspective aims to uncover systemic risk patterns from historical accident data. For example, Wang et al. [5], using accident case data, applied semantic networks and word frequency statistics to identify key risk factors in subway operation accidents. Zhang et al. [6] conducted statistical analyses of accident data to examine the patterns of subway incidents in Shanghai. In addition, network models have also been adopted to study subway accidents, primarily from the perspective of network topology structure [7]. However, these approaches face limitations in analyzing complex human and organizational factors, and they are inadequate for uncovering the deeper causes of subway operation accidents. Causation models, as an important tool for accident analysis, offer a systematic and multidimensional means of identifying critical factors involved in accidents, which can, to some extent, compensate for the aforementioned limitations. They provide a more comprehensive understanding of accident mechanisms and have been widely applied in the analysis of hazardous chemical accidents [8–10], coal mine disasters [11–13], power system failures [14–16], and construction site accidents [17–19]. In the field of subway operational accident research, He et al. analyzed the sources of operational risks using accident models such as fault tree analysis, and further identified rolling stock system failures and signal-communication system failures as the most critical risk factors through data envelopment analysis [20]. Wang et al. identified risk factors associated with subway operation accidents using accident models such as AcciMap, thereby providing support for constructing an accident semantic network [4]. As such, causation models are gradually emerging as a key method in the study of subway operation accidents. Therefore, this study adopts a causation model approach to identify risk factors in subway operation accidents.

In the field of risk research, Wang et al. [7] developed an subway risk network model based on accident case studies. Derrible and Kennedy [21] investigated the vulnerability of network lines across 33 global metro systems using complex network theory. Lee and Hur [22] applied numerical simulation methods to analyze the risk of subway fire accidents. Complex network theory has been increasingly utilized in the study of risk evolution within subway networks [23–25], and its applications have extended to the analysis of risks in electrical accidents [26,27], construction incidents [28,29], and chemical industry accidents [30,31]. Compared to traditional risk assessment methods such as fault tree analysis and event tree analysis, complex networks offer distinct advantages in visualizing system structures, analyzing the intricate coupling relationships among risk factors, and identifying critical risk elements. Given that subway systems are inherently complex and their operational safety is influenced by a multitude of interconnected and interdependent factors, it is essential to explore the coupling mechanisms among various risk factors contributing to operational accidents. Such an investigation can help clarify the underlying formation mechanisms of these accidents. This, in turn, will support the development of more targeted and effective risk management strategies for subway operations. Therefore, this study employs complex network methods to identify key risk factors associated with subway operation accidents.

Building upon the aforementioned research, this study integrates causation models, association rule mining, and complex network theory to construct a comprehensive analytical framework for identifying risk factors in subway operation accidents. The proposed model aims to uncover the key risk factors contributing to such accidents, thereby providing a theoretical foundation for enhancing the safety management of subway operations. A safe and reliable subway operational system is the prerequisite and cornerstone for achieving the sustainability goals of urban public transportation, such as environmental friendliness, economic efficiency, and social inclusiveness. This study, through data-driven analysis of key risk factors, aims to enhance the inherent resilience of the operational system. This is of significant importance for ensuring the long-term safety and stability of subway operations and promoting the sustainable development of subway systems.

## 2. Materials and methods

### 2.1. Data sources

Accident cases can reveal the coupling mechanisms among risk factors and the evolutionary patterns of accidents, providing critical data support for risk factor analysis. To enhance the quality of accident data and ensure comprehensiveness and diversity, this study collected 76 subway operation accident cases that occurred in China between 2009 and 2024. The geographical and temporal scope of this study was determined by considering the comparability of accident data, the development trajectory of China’s urban rail transit, and data completeness. Restricting the analysis to China helps maintain the comparability of accident cases under relatively consistent accident-reporting practices, regulatory frameworks, and accident classification standards, thereby reducing data heterogeneity that could affect risk-factor identification and network construction. Previous studies have shown that China’s urban rail transit entered a period of rapid and large-scale development after 2008, with substantial growth in operating scale and the number of cities with operational systems during 2008–2015 [32]. After 2015, an increasing number of cities gradually transitioned from single-line operation to multi-line network operation, accompanied by continued advances in system automation and intelligence. Therefore, the period from 2009 to 2024 covers an important development process of China’s urban rail transit from rapid system expansion to large-scale networked operation, providing a broad empirical basis for identifying accident risk factors and their associations under different stages of operational development and operating environments. The year 2024 was selected as the endpoint because it was the most recent complete calendar year for which relatively comprehensive accident information was available when data collection was conducted; using a complete calendar year also helps reduce the risk of data truncation and sample omission caused by incomplete disclosure of more recent accident information. The cases were gathered from multiple sources, including official websites of provincial and municipal governments of the People's Republic of China, emergency management department portals, news reports, and published books. The accidents collected in this study occurred across 16 cities in China. Among them, the top three cities with the highest number of accidents—Beijing, Shenzhen, and Guangzhou—reported a total of 36 incidents, accounting for 47.37% of all cases. According to national standards and regulatory documents, including the Classification and Coding of Production Safety Accidents [33] and the Administrative Measures for Information Reporting and Analysis of Urban Rail Transit Operational Hazardous Incidents [34], the accident case database covered 13 categories of accident types, including train collision, train derailment, train conflict, vehicle-related injury, falls from height, and electric shock. In addition, the database also included severe accident consequence types such as vehicle damage, operation interruption, train delay, and casualties. The distribution of the specific accident types among the 76 accidents is presented in Table 1.

**Table 1:** Distribution of the 76 subway operation accidents by accident type.

No.	Accident type	Number	Proportion
1	Train collision	20	0.26
2	Train derailment	12	0.16
3	Train conflict	8	0.11
4	Others	8	0.11
5	Flooding and backflow intrusion in stations and track areas	6	0.08
6	Falls from height	6	0.08
7	Major signaling system failure	4	0.05
8	Vehicle-related injury	2	0.03
9	Major vehicle failure	2	0.03
10	Electric shock	2	0.03
11	Catenary breakage or collapse	2	0.03
12	Hoisting-related injury	2	0.03
13	Severe defects in civil structures and foundations	2	0.03

**Note:** The 76 cases comprise subway operation accidents collected in China between 2009 and 2024. Proportion represents the number of cases in each accident category divided by the total number of cases.

Although the 76 accident cases used in this study may not constitute a large sample size, they remain reasonably adequate within the framework of the present research. First, subway operation accidents are characterized as low-frequency but high-consequence events. Unlike high-incidence categories such as construction accidents and road traffic accidents, subway systems possess a relatively high degree of safety redundancy. Consequently, the number of accessible accident cases that can support in-depth coding and analysis based on the 24Model is inherently limited, and this study collected such cases to the greatest extent possible. Second, this study adhered to the principle of prioritizing data quality over quantity. The 24Model requires accident reports to contain sufficiently detailed information regarding the accident process, causation mechanisms, and related circumstances to support accident causation analysis. Blindly expanding the sample size by including incomplete cases, such as incidents documented only through brief news reports, could introduce substantial noise and thereby compromise the accuracy of data extraction. Despite the limited sample size, previous studies with comparable datasets have demonstrated the applicability and validity of similar approaches. For example, Su et al. [35] employed the Apriori algorithm based on 72 coal mine accidents to reveal the association relationships among coal mine safety risk factors and identify key risk factors. Wang et al. [36] extracted 418 strong association rules from 106 hazardous chemical accidents using the Apriori algorithm and subsequently utilized these rules as the topological structure of a Bayesian network model. Wu et al. [37] investigated the interaction relationships among subway construction safety risk factors based on 101 subway construction accident reports using association rule mining and complex network methods. Therefore, the sample size adopted in this study can be considered reasonably acceptable for supporting the methodological framework of the present research.

### 2. 2. Research framework

**Fig 2:**
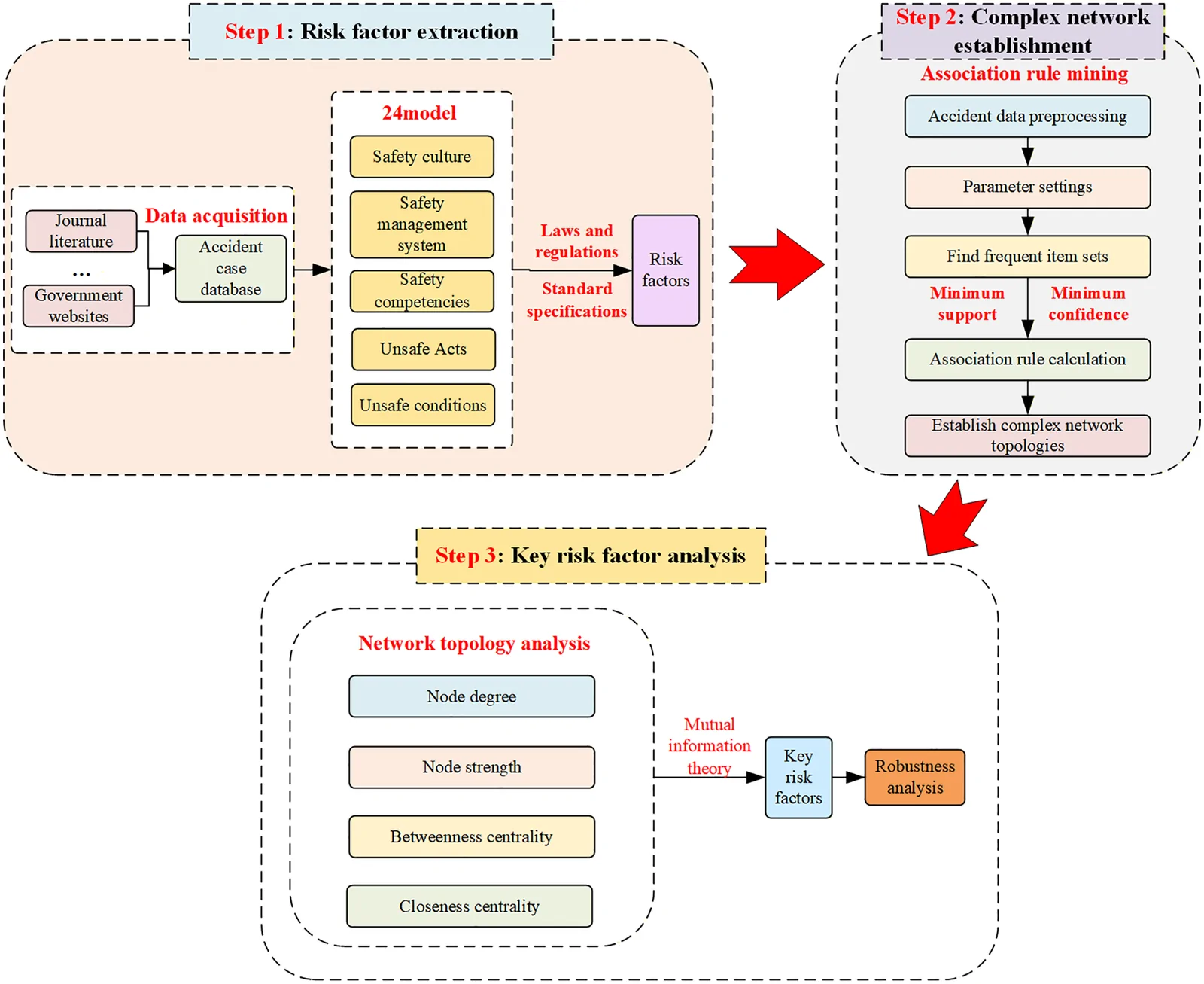
Research framework. The framework includes three stages: risk-factor extraction using the 24Model and relevant regulatory documents, complex-network construction through association rule mining, and identification of key risk factors using network topology indicators and mutual information.

The proposed methodology consists of three main components, as illustrated in Fig 2. The first component involves the extraction of risk factors. Based on accident reports, risk factors are identified using the 24Model in combination with relevant laws, regulations, and technical standards. The second component focuses on the construction of a complex network. Using the established accident case database, association rule mining is applied to determine the relationships among risk factors. Risk factors are represented as network nodes, and their associations are represented as edges, thereby constructing the network's topological structure. The third component is complex network analysis. Key risk factors are identified through various analytical metrics, including node degree, node strength, and mutual information theory.

### 2.3. Accident causation model

**Fig 3:**
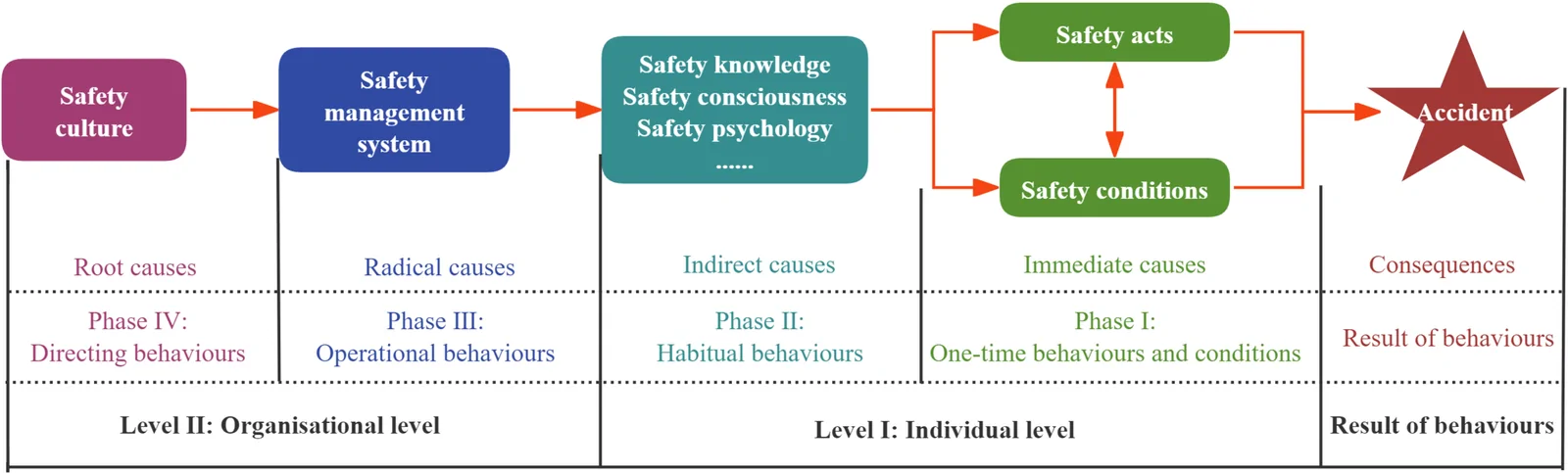
24Model. The model links organizational-level causes, including safety culture and the safety management system, with individual-level indirect and immediate causes, which ultimately lead to accident consequences.

Accident causation models are theoretical frameworks distilled from extensive accident investigations and analyses. These models reflect the underlying mechanisms of accidents and serve as essential tools for accident prevention, control, and root cause analysis. The 24Model is a theoretical framework for accident causation. It categorizes the causes of accidents into two hierarchical levels—organizational factors (including safety culture and safety management systems) and individual factors (including habitual behaviors, one-off behaviors, and physical conditions)—across four stages. This model is widely used for both accident analysis and accident prevention. The 24Model integrates the strengths of several classical theories, including Heinrich’s Domino Theory [38], Bird’s Linear Causation Model, Stewart’s MMOS Model, and Reason’s Swiss Cheese Model [39]. It has been widely applied in various high-risk industries such as chemical manufacturing [40,41], transportation [42,43], and aviation [44]. The model is particularly effective in identifying deep-rooted organizational causes, such as defects in safety management system and safety culture, which contribute to accident occurrence. The 24Model was selected because its hierarchical structure closely matches the multi-level causal information contained in subway operation accident reports. It organizes accident causes from immediate unsafe acts and unsafe conditions to defects in individual safety capabilities, safety management systems, and safety culture. This structure enables the analysis not only to identify visible failures at the operational front line but also to trace the latent organizational conditions that allowed these failures to occur. In addition, its clearly defined categories and relatively simple structure facilitate the consistent coding of heterogeneous narrative accident reports into standardized risk factors, which is essential for the subsequent association rule mining and complex network construction. By linking individual behaviors with organizational control defects, the model also allows the identified causes to be translated into targeted measures involving training, procedures, supervision, safety management systems, and safety culture. Therefore, this study adopts the 24Model to identify risk factors associated with operational accidents in subway systems, as illustrated in Fig 3. Given the need to identify deep-seated and systematic risk factors that align with the 24Model theoretical framework from complex subway operational accident reports, this task goes beyond simple keyword matching or surface-level semantic analysis. It requires analysts to possess a profound understanding of the 24Model, rail transit operational safety management, and accident causation analysis. Therefore, the risk factor identification and analysis in this study were primarily carried out by experts with associate professor-level or higher academic titles, who are familiar with the 24Model theory and have more than five years of research experience in subway operational safety management.

### 2.4. Association rule mining

Association rule mining is a machine learning algorithm that discovers potential relationships in data by extracting frequent itemsets. It has been widely applied in various domains such as medicine [45], the internet [46], and safety management [47,48]. The Apriori algorithm is one of the most classical algorithms for mining association rules among factors. It identifies frequent itemsets through a level-wise search process based on minimum support and minimum confidence thresholds [49]. Let the database be *D*={*T*_1_, *T*_2_,  ..., *T*_*n*_}, where each *T*_*i*_ is a set of distinct items. Let *I* = {*i*_1_, *i*_2_,  ..., *i*_*m*_} denote the set of all items. An association rule takes the form *X*⇒*Y,* where *X* ∈ *I, Y* ∈ *I, and X*∩*Y=*∅*.*

The support of the rule *X*⇒*Y* in the database *D* is defined as support (*X*⇒*Y*), which represents the probability that *X* and *Y* occur simultaneously, as shown in Equation (1):


$$\begin{array}{c} \text{support}(X\Rightarrow Y)=P(X\cup Y)=\frac{\text{count}(X\cup Y)}{\left|D\right|} \end{array}$$ (1)

The confidence of the rule *X*⇒*Y* in the database *D* is defined as confidence(X⇒Y), which represents the conditional probability that *Y* occurs given that *X* has occurred, as shown in Equation (2):


$$\begin{array}{c} \text{confidence}(X\Rightarrow Y)=P\left(X|Y\right)=\frac{\text{support}(X\cup Y)}{\text{support}(X)} \end{array}$$ (2)

Lift measures the strength of the association between the antecedent *X* and consequent *Y* relative to statistical independence, as shown in Equation (3):


$$\begin{array}{c} \text{Lift}(X\to Y)=\frac{\text{Confidence}(X\to Y)}{\text{Support}(Y)}=\frac{P(X\cap Y)}{P(X)P(Y)} \end{array}$$ (3)

A lift value greater than 1 indicates a positive association between *X* and *Y*, whereas a value equal to 1 indicates statistical independence. In this study, lift was used as the weight of the corresponding directed edge to represent the relative strength of the association between risk factors.

In this study, IBM SPSS Modeler 18.0 was used to conduct Apriori-based association rule mining. Based on the support and confidence measures defined above, the Apriori algorithm was applied to identify associations among the risk factors. The resulting association rules provided the relational information required for constructing the complex network structure in the subsequent analysis.

### 2.5. Network construction and analysis

A complex network is a specialized type of network structure that models a complex system by abstracting its components as nodes and the relationships between them as edges [28]. In this study, complex network theory is applied to analyze risk factors associated with subway operation accidents. Based on the association rules among risk factors, a weighted and directed complex network is constructed. By analyzing the importance of nodes within the network, this approach enables the exploration of accident evolution patterns in subway systems [50,51], thereby facilitating the identification of key risk factors.

#### 2.5.1. Directed weighted network construction.

In the constructed network, nodes represent risk factors of subway operation accidents, while directed edges represent the association rules between risk factors mined using the Apriori algorithm, with the lift of each rule serving as the weight of the corresponding edge. A directed and weighted network is established, denoted as DiG=(N, DiE). The network structure can be represented by an adjacency matrix G, as shown in Eqs (4)–(5):


$$\begin{array}{c} \text{G}=\begin{array}{cc} \begin{array}{c} \begin{array}{c} {\text{n}}_{\text{1}} \end{array}\\ \begin{array}{c} {\text{n}}_{\text{2}}\\ \begin{array}{c} \cdots \\ {\text{n}}_{\text{p}} \end{array} \end{array} \end{array} & \begin{array}{c} \begin{array}{cc} {\text{n}}_{\text{1}} & \begin{array}{cc} {\text{n}}_{\text{2}} & \begin{array}{cc} \cdots & {\text{n}}_{\text{p}} \end{array} \end{array} \end{array}\\ \left[\begin{array}{ccc} \begin{array}{cc} \begin{array}{c} {\text{g}}_{\text{11}}\\ {\text{g}}_{\text{21}} \end{array} & \begin{array}{c} {\text{g}}_{\text{12}}\\ {\text{g}}_{\text{22}} \end{array} \end{array} & \begin{array}{c} \cdots \\ \cdots \end{array} & \begin{array}{c} {\text{g}}_{\text{1p}}\\ {\text{g}}_{\text{2p}} \end{array}\\ \begin{array}{cc} \cdots & \cdots \end{array} & \cdots & \cdots \\ \begin{array}{cc} {\text{g}}_{\text{p1}} & {\text{g}}_{\text{p2}} \end{array} & \cdots & {\text{g}}_{\text{pp}} \end{array}\right] \end{array} \end{array} \end{array}$$ (4)

$$\begin{array}{c} {\text{g}}_{\text{ij}}=\left\{ \begin{array}{c} {\text{w}}_{\text{ij}}{\text{e}}_{\text{ij}}\text{ if i points to j }\\ \text{0}\text{ otherwise} \end{array}\right. \end{array}$$ (5)

Where ${\text{g}}_{\text{ij}}$ represents the connection from node nᵢ to node n_j_; ${\text{w}}_{\text{ij}}$ denotes the weight of the directed edge from nᵢ to nⱼ, which corresponds to the confidence level of the association rule i ⇒ j; ${\text{e}}_{\text{ij}}$ indicates whether there is a directed edge from n_i_ to n_j_, where ${\text{e}}_{\text{ij}}$ = 1 if connected and ${\text{e}}_{\text{ij}}$ = 0 otherwise.

#### 2.5. 2. Node degree.

Node degree refers to the total number of edges connected to a given node. In a directed network, node degree can be classified into three types: total degree, out-degree, and in-degree. The corresponding formulas are as shown in Eqs (6)–(8):


$$\begin{array}{c} k_{i}^{in}=\sum_{j,j\text{≠}i}e_{ij} \end{array}$$ (6)

$$\begin{array}{c} k_{i}^{out}=\sum_{j,j\text{≠}i}e_{ij} \end{array}$$ (7)

$$\begin{array}{c} k_{i}^{total}=k_{i}^{in}+k_{i}^{out} \end{array}$$ (8)

Where ${\text{k}}_{\text{i}}^{in}$ denotes the in-degree of node ni; ${\text{k}}_{\text{i}}^{\text{out}}$ denotes the out-degree of node ni; and ${\text{k}}_{\text{i}}^{\text{total}}$ denotes the total degree of node ni.

#### 2.5.3. Node strength.

Node strength refers to the sum of the weights of all edges connected to a given node. In a directed network, node strength can be categorized into three types: total strength, out-strength, and in-strength. The corresponding formulas are as shown in Eqs (9)–(11):


$$\begin{array}{c} S_{i}^{in}=\sum_{j,j\text{≠}i}w_{ij}e_{ij} \end{array}$$ (9)

$$\begin{array}{c} S_{i}^{out}=\sum_{j,j\text{≠}i}w_{ij}e_{ij} \end{array}$$ (10)

$$\begin{array}{c} S_{i}^{total}=S_{i}^{in}+S_{i}^{out} \end{array}$$ (11)

Where $S_{i}^{in}$ denotes the in-strength of node *ni*; $S_{i}^{out}$ denotes the out-strength of node *ni*; and $S_{i}^{total}$ denotes the total strength of node *ni*.

#### 2.5.4. Betweenness centrality.

Betweenness Centrality. Betweenness centrality of a node is defined as the number of shortest paths in the network that pass through the node. It reflects the node’s role as a hub and its capacity for information transfer within the network. The calculation formula is as shown in Eqs (12)–(13):


$$\begin{array}{c} B_{v}=\sum_{\begin{array}{c} \forall i,j\in N\\ i\text{≠}v\text{≠}j \end{array}}\left(\frac{\sigma_{ij}(v)}{\sigma_{ij}}\right) \end{array}$$ (12)

$$\begin{array}{c} {BC}_{v}=\frac{B_{v}}{\left(p-\text{1})(p-\text{2}\right)} \end{array}$$ (13)

where $\sigma_{ij}$ denotes the total number of shortest paths from node *n*_*i*_ to *n*_*j*_; $\sigma_{\text{ij}}(\text{v})$ denotes the number of shortest paths from node *n*_*i*_ to node *n*_*j*_ that pass through node *v*; *N* denotes the set of nodes in the network; *p* denotes the total number of nodes; and $B_{v}$ and ${BC}_{v}$ denote the betweenness and normalized betweenness centrality of node vvv, respectively.

#### 2.5.5. Mutual-information-based node importance.

This study employs a node importance ranking algorithm based on mutual information theory. This method takes into account both the connectivity strength between network nodes and the network’s topological characteristics. The calculation formula is as shown in Eqs (14)–(15):


$$\begin{array}{c} I_{ij}=\left\{ \begin{array}{c} \text{ln}\frac{S_{i}^{out}}{S_{j}^{in}},e_{ij}=\text{1}\;\\ \text{0},e_{ij}=\text{0} \end{array}\right. \end{array}$$ (14)

$$\begin{array}{c} I(i)=\sum_{m\text{≠}i}I_{im}-\sum_{n\text{≠}i}I_{ni} \end{array}$$ (15)

Where $S_{i}^{in}$ denotes the in-strength of node *ni*; $S_{i}^{out}$ denotes the out-strength of node *ni*; and $S_{i}^{total}$ denotes the total strength of node *ni*. where $S_{i}^{out}$ denotes the out-strength of node *i*; $S_{j}^{in}$ denotes the in-strength of node *j*; $e_{ij}$ indicates whether a directed edge exists from node *i* to node *j*, with $e_{ij}$=1 when the edge exists and $e_{ij}$=0 otherwise; $I_{ij}$ denotes the information contribution from node *i* to node *j*; and I(i) denotes the importance score of node *i*. A higher I(i) indicates a greater relative information contribution and systemic importance of the corresponding risk factor within the network.

## 3. Results

### 3.1. Accident risk factor identification

Within the 24Model framework, individual-level causal factors are categorized into unsafe acts and unsafe conditions. The unsafe acts are primarily committed by personnel of subway operating organizations and are classified according to job roles, including drivers, dispatchers, station attendants, and maintenance workers etc. Based on the analysis of accident case studies, this study identifies eight types of unsafe human behaviors, as shown in Table 2. A regulatory document for the safety of subway operations issued in China [52] classifies equipment and facilities into subsystems: vehicle systems, power supply systems, track and line systems, electromechanical systems, communication systems, signaling systems, and civil infrastructure etc. Based on accident case analysis, this study identifies six categories of unsafe physical conditions, as shown in Table 2.

**Table 2:** Risk factors of subway operation accidents identified using the 24Model and their corresponding codes.

Unsafe acts	Dispatchers illegal operation D1	Drivers illegal operation D2	Maintenance personnel illegal operation D3
	Construction workers illegal operation D4	Station attendants illegal operation D5	Designers illegal operation D6
	Unauthorized design modifications D7	The absence of unified on-site command and dispatch by leadershipD8	
Unsafe conditions	Power supply systems failures D9	Drainage systems failures D10	Civil infrastructure failures D11
	Signaling systems failures D12	Track and line systems failures D13	Vehicle systems failures D14
Insufficient safety competencies	Insufficient security knowledge C1	Insufficient safety consciousness C2	Luck-driven mentality C3
	Risk-taking mentality C4		
Safety management system defects	Unclear safety responsibilities for employees B1	Failure to effectively implement the all-staff safety responsibility system B2	Failure to implement shift handover procedures B3
	Absence of operational procedures B4	Inadequate enforcement of safety supervision and inspection systems B5	Lack of detailed train dispatching regulations B6
	Failure to follow construction operation procedures B7	Insufficient coordination capacity in dispatch command B8	Failure to follow dispatch operation procedures B9
	Failure to follow train operation procedures B10	Lack of safety training B11	Lack of targeted content in safety training B12
	Failure to enforce certification-based job qualification B13	Ineffective safety training outcomes B14	Inadequate identification of safety risks B15
	Failure to inform employees of existing safety risks and countermeasures B16	Absence of a safety risk management mechanism B17	Blind spots in hidden danger identificationB18
	Failure to promptly and effectively rectify identified hidden danger B19	Failure to carry out hidden danger identification B20	Absence of a hidden danger identification and mitigation systemB21
	Incomplete power supply system B22	Inadequate flood protection capability of safety equipment and facilities B23	Failure to conduct risk assessment for equipment maintenance operations B24
	Absence of a maintenance operation plan B25	Inadequate maintenance of equipment and facilities B26	Incomplete signaling system B27
	Emergency response plan not approved through formal review B28	Failure to conduct regular emergency drills B29	Insufficient content in the emergency response plan B30
	Inadequate coordination across emergency response plans B31	Emergency response plan not updated in a timely manner B32	Absence of emergency response plans B33
	Inadequate content in the on-site response plan B34	Absence of an on-site response plan B35	Failure to promptly report accident information B36
	Failure to evacuate passengers in a timely manner B37	Failure to issue timely suspension instructions for the rail network B38	Failure to activate emergency response B39
	Insufficient safety oversight of subcontractors B40	Inadequate on-site safety inspection B41	Insufficient supervision of on-site operations B42
	Absence of a feasible construction plan B43	Failure to complete approval procedures B44	Failure to carry out technical disclosure B45
	Failure to set up safety warning signs B46	Missing documentation of on-site operational procedures B47	Poor quality control in construction project execution B48
Safety culture defects	Safety importance A1	Beliefs of zero-accident A2	Safety and management integration A3
	Safety consciousness A4	Primary responsibility for workplace safety A5	Safety responsibility of managers A6
	Individual involvement in safety A7	Demand of safety training A8	Responsibilities for safety in every department A9
	Enforcement of safety rules A10	Workplace audits and inspections A11	Facility satisfaction A12
	Safety of contractors and subsidiaries A13	Emergency capability A14	

**Note:** Risk factors are classified according to the 24Model into safety culture defects (A), safety management system defects (B), insufficient safety competencies (C), and one-time unsafe acts and unsafe conditions (D). The alphanumeric codes are used to identify the corresponding risk factors in the subsequent association-rule and complex-network analyses.

Individual safety capability factors primarily include safety knowledge, safety consciousness, and safety psychology. Safety knowledge refers to an individual’s understanding, mastery, and practical application of professional knowledge, operational experience, safety regulations, and safety skills related to subway operations. When employees lack sufficient safety knowledge, they may be unable to identify potential hazards in the workplace, lack the skills and competence to respond to risks, and thereby face a higher likelihood of accident occurrence. Safety consciousness refers to an employee’s ability to perceive risks and respond to them in a timely manner. When safety consciousness is inadequate, employees may fail to recognize hazards in their surroundings and be unable to implement effective emergency responses when accidents occur. Safety psychology has a significant influence on individual safety behavior, primarily manifested in two forms: luck-driven mentality and risk-taking mentality. The luck-driven mentality is a major inducement of rule-breaking behavior, while a risk-taking mentality often leads to a lack of necessary vigilance during operations, thereby increasing the probability of accidents.

The safety management system can influence unsafe acts both indirectly by shaping individual safety capabilities and directly by regulating operational behavior. According to the regulatory document for the safety of subway operations issued in China [52], accident case studies were analyzed from the perspectives of safety responsibility systems, safety regulations, safety training, risk management, hazard identification and mitigation, emergency response, emergency handling, and construction operation management. A total of 48 types of safety management system defects were identified, as shown in Table 2.

In the 24Model framework, safety culture is regarded as the ideological foundation that guides safety work and is often referred to as the safety philosophy. Safety culture manifests through multiple factors closely associated with the occurrence of accidents. It exerts a direct influence on the guiding behavior of the safety management system and also indirectly affects individual actions by shaping personal safety capabilities. It has been recognized as one of the key factors influencing unsafe acts. Based on the 32 specific elements of safety culture defined in the 24Model [53], this study analyzes accident case studies in the context of subway operations. The findings indicate that defects in safety culture are mainly concentrated in 14 elements, as shown in Table 2.

### 3. 2. Associations among risk factors

IBM SPSS Modeler was employed to mine association rules related to risk factors in subway operation accidents. The thresholds were set as follows: support ≥ 0.1 and confidence ≥ 0.1. There is no universally accepted standard for determining the minimum support threshold and minimum confidence threshold in association rule mining, as their selection should align with the specific research objectives. In this study, relatively low threshold values were adopted to maximize the identification of potential risk couplings, including low-frequency but potentially high-value association rules. Meanwhile, sensitivity analysis was conducted by increasing the minimum support threshold and minimum confidence threshold. The results indicated that when the minimum support threshold was set to 0.1 and the minimum confidence threshold was set to 0.2, the number of association rules decreased from 609 to 404. However, all key nodes remained present, and the core network structure did not exhibit significant changes. When the minimum support threshold was further increased to 0.15 while maintaining the minimum confidence threshold at 0.2, the number of association rules further decreased to 271. Under this condition, key nodes B1 and A6 became isolated nodes, and the core network structure changed significantly. Therefore, in order to avoid overlooking valuable but low-frequency association rules, this study ultimately adopted a minimum support threshold of 0.1 and a minimum confidence threshold of 0.1, thereby retaining low-frequency yet potentially high-value association rules.

Prior to conducting association rule analysis among risk factors, 76 collected accident cases in unstructured text format were converted into a format suitable for Apriori analysis. The accident investigation reports were analyzed by experts familiar with the 24Model and subway operational safety management. If explicit textual evidence or direct inference of the presence of a specific risk factor was found in the report, a “1” was assigned to indicate the presence of that risk factor in the accident; otherwise, a “0” was used to indicate its absence, as shown in Table 3. For example, an investigation report on a metro rear-end collision accident stated that “the chief dispatcher improperly instructed the on-duty dispatcher to release the detention of Train 032, failed to report the situation in accordance with the prescribed job responsibilities, and did not inform the driver of Train 032 of a temporary stop ahead.” In addition, “under snowy conditions, the driver of Train 032 did not apply low-level braking; after the signaling system issued a deceleration prompt, no braking action was taken. Following the second deceleration prompt from the signaling system, braking was applied after 1.4 s; 0.2 s later, the speed of Train 032 exceeded the threshold for triggering emergency braking, thereby causing the signaling system to initiate emergency braking.” Based on this information, experts determined that two risk factors—dispatchers’ illegal operation and drivers’ illegal operation—were present in this accident. The above approach represents a common method for the quantitative investigation of accident risks based on the textual information contained in accident investigation reports. For instance, Fu et al. collected 126 metro deep foundation pit construction accident reports and extracted the causes from each report to form a standardized accident dataset for association rule mining, ultimately identifying 32 strong association rules among factors and 73 strong association rules between factors and accidents [54]. Wang et al. manually extracted risk factors and the relationships among factors from 201 subway operation accident investigation reports, constructed a risk network, and analyzed key risk factors [7]. Moreover, methods that combine expert judgment with the textual information of accident investigation reports to determine the digital representation of risk factors have been widely applied in accident risk studies in the construction [55,56], chemical [36], and coal mining [57] industries. To ensure the reliability of the risk factor dataset, Cohen’s kappa coefficient was employed to assess the inter-rater consistency of the judgments made by each expert. The results indicate that the average kappa value for the 80 factors was 0.72, demonstrating good inter-rater reliability in the identification of risk factors in this study. Two experts independently identified and coded the risk factors in each accident report. In cases where inconsistencies arose in the coding results, the two experts first attempted to reach a consensus through discussion and consultation. If disagreement still remained after deliberation, a third expert with an associate senior title or above in the field of subway operation safety research was invited to conduct arbitration, and the arbitration result was adopted as the final coding outcome. After computation, and following the removal of rules inconsistent with the logical framework between different levels in 24Model and expert knowledge, a total of 609 valid association rules were obtained. A selection of the mined rules is presented in Table 4.

**Table 3:** Partial binary coding matrix of risk factors extracted from the subway operation accident cases.

No.	C1	C2	C3	C4	D1	D2	D3	D4	D5	D6	D7	D8	D9	D10	D11	···	A14
1	1	0	1	0	1	1	0	0	0	0	0	0	0	0	0	···	0
2	0	1	1	0	0	0	1	0	0	0	0	0	1	0	0	···	1
3	1	1	0	1	1	1	0	0	0	0	0	0	0	0	0	···	1
4	0	1	1	0	0	0	0	1	0	0	0	0	0	1	0	···	0
5	1	0	1	0	0	0	0	1	0	0	1	0	0	1	1	···	0
6	0	1	1	0	0	0	0	1	0	0	0	0	0	0	0	···	0
7	0	1	0	1	0	0	0	1	0	0	0	0	0	0	0	···	0
8	0	1	1	0	0	0	0	1	0	0	0	0	0	1	0	···	0
9	1	1	0	1	0	0	0	1	0	0	0	0	0	0	0	···	0

**Note:** Each row represents one accident case, and each column represents a risk factor coded according to Table 2. A value of 1 indicates that the corresponding risk factor was identified in the accident report, whereas 0 indicates that it was not identified. Only a portion of the complete coding matrix is shown.

**Table 4:** Selected association rules among subway operation accident risk factors identified using the Apriori algorithm.

No.	Association rules	Support	Confidence	Lift
1	Failure to follow construction operation procedures→Construction workers illegal operation	0.29	0.91	2.03
2	Inadequate enforcement of safety supervision and inspection systems→Insufficient safety consciousness	0.45	0.88	1.29
3	Insufficient supervision of on-site operations→Failure to follow construction operation procedures	0.18	0.86	2.96
4	Failure to effectively implement the all-Insufficient safety consciousness	0.34	0.85	1.24
5	Lack of targeted content in safety training→Insufficient security knowledge	0.16	0.83	2.64
6	Inadequate on-site safety inspection →Construction workers illegal operation	0.29	0.73	1.63
7	Failure to effectively implement the all-staff safety responsibility system→Inadequate enforcement of safety supervision and inspection systems	0.34	0.69	1.55
8	Failure to promptly and effectively rectify identified hidden danger →risk-taking mentality	0.16	0.67	2.30
9	Inadequate enforcement of safety supervision and inspection systems→Ineffective safety training outcomes	0.45	0.65	1.54
10	Inadequate identification of safety risks → Blind spots in hidden danger identification	0.29	0.64	1.86

**Note:** Association rules are expressed as antecedent → consequent. Support represents the joint occurrence probability of the antecedent and consequent, confidence represents the conditional probability of the consequent given the antecedent, and lift indicates the strength of association relative to statistical independence. The minimum support and confidence thresholds were both set to 0.1. Only selected rules are presented; the complete set of valid association rules is provided in S1 File.

Based on the association rule mining results derived from 76 subway operation accident cases, a complex network topology model was constructed. In this model, the antecedents and consequents of the association rules were treated as nodes in the network, and the association relationships between them were represented as directed edges. The lift level of each rule was used as the weight of the corresponding directed edge. This modeling approach allows for a more accurate representation of the network structure and provides a foundation for further analysis of complex subway accident risk factors using complex network methodologies.

### 3.3. Overall structure of the risk-factor network

**Fig 4:**
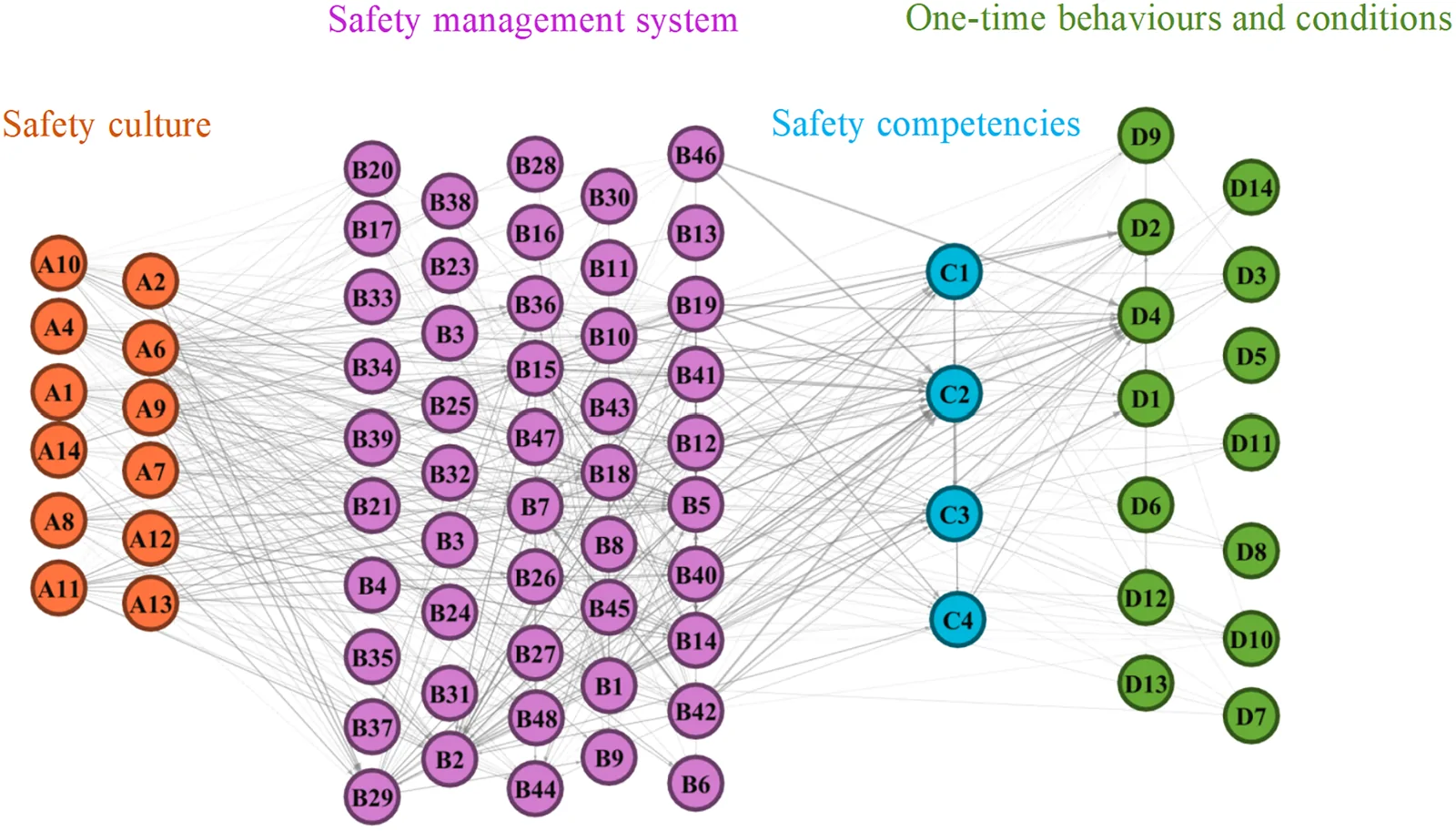
Subway operation accidents risk factors network. The network comprises 78 risk-factor nodes and 609 directed edges derived from the association rules mined from the accident case database described in this study. Node colors denote the four risk-factor categories of the 24Model: safety culture (A, orange), safety management system (B, purple), safety competencies (C, blue), and one-time behaviors and conditions (D, green). A directed edge represents an association rule from an antecedent risk factor to a consequent risk factor, and the edge weight represents lift. The network was visualized using Gephi.

The objective of constructing and analyzing the complex network of subway operation accidents is to quantitatively identify the key risk factors, thereby enabling the development of more targeted accident prevention strategies. Based on the association rules mined in Section 3.2, a weighted network consisting of 78 nodes and 609 directed edges was constructed. The risk factor network model of subway operation accidents was then visualized using Gephi software, as illustrated in Fig 4.

To explore the interaction characteristics among risk factors in subway operation accidents, the topological features of the constructed complex network were analyzed. The analysis included network-level indicators such as the number of nodes and edges, clustering coefficient, average path length, and network density, as well as node-level indicators such as node degree, node strength, betweenness centrality, and closeness centrality. These measures were calculated according to their conventional definitions in complex-network analysis. A higher clustering coefficient indicates stronger local clustering among nodes, a shorter average path length indicates greater overall network accessibility, network density represents the proportion of realized connections relative to all possible connections, and closeness centrality reflects how readily a node can reach other nodes through shortest paths. The corresponding results are summarized in Table 5 and provide a basis for further characterizing the structural properties of the risk-factor network.

**Table 5:** Global and node-level topological characteristics of the subway operation accident risk-factor network.

Topology index	Numeric value	Topology index	Numeric value	Topology index	Numeric value
Number of nodes	78	Maximum node degree	55	Maximum betweenness centrality	236.82
Number of edges	609	Minimum node degree	1	Minimum betweenness centrality	0
Clustering coefficient	0.392	Maximum strength of node	203.98	Maximum closeness centrality	1
Average path length	1.829	Minimum strength of node	3.17	Minimum closeness centrality	0
Network density	0.101				

**Note:** The network comprises 78 risk-factor nodes and 609 directed edges. Network-level characteristics include the clustering coefficient, average path length, and network density, whereas node-level characteristics include degree, node strength, betweenness centrality, and closeness centrality.

As shown in Table 5, the average clustering coefficient of the subway operation accident network is 0.392, indicating a high level of clustering. This suggests that nodes in the network are closely connected and more likely to exert rapid cross-influences on one another. The average path length is 1.829, implying that any two non-adjacent nodes in the network can be connected through relatively short paths. This means that even indirectly related risk factors may influence each other through short chains of reactions. The network density is 0.101, which is significantly lower than 1, indicating that the network is relatively sparse, and most nodes are not directly connected. Therefore, identifying key risk factors is crucial for effectively preventing accidents.

### 3.4. Identification of key risk factors in subway operation accidents

**Fig 5:**
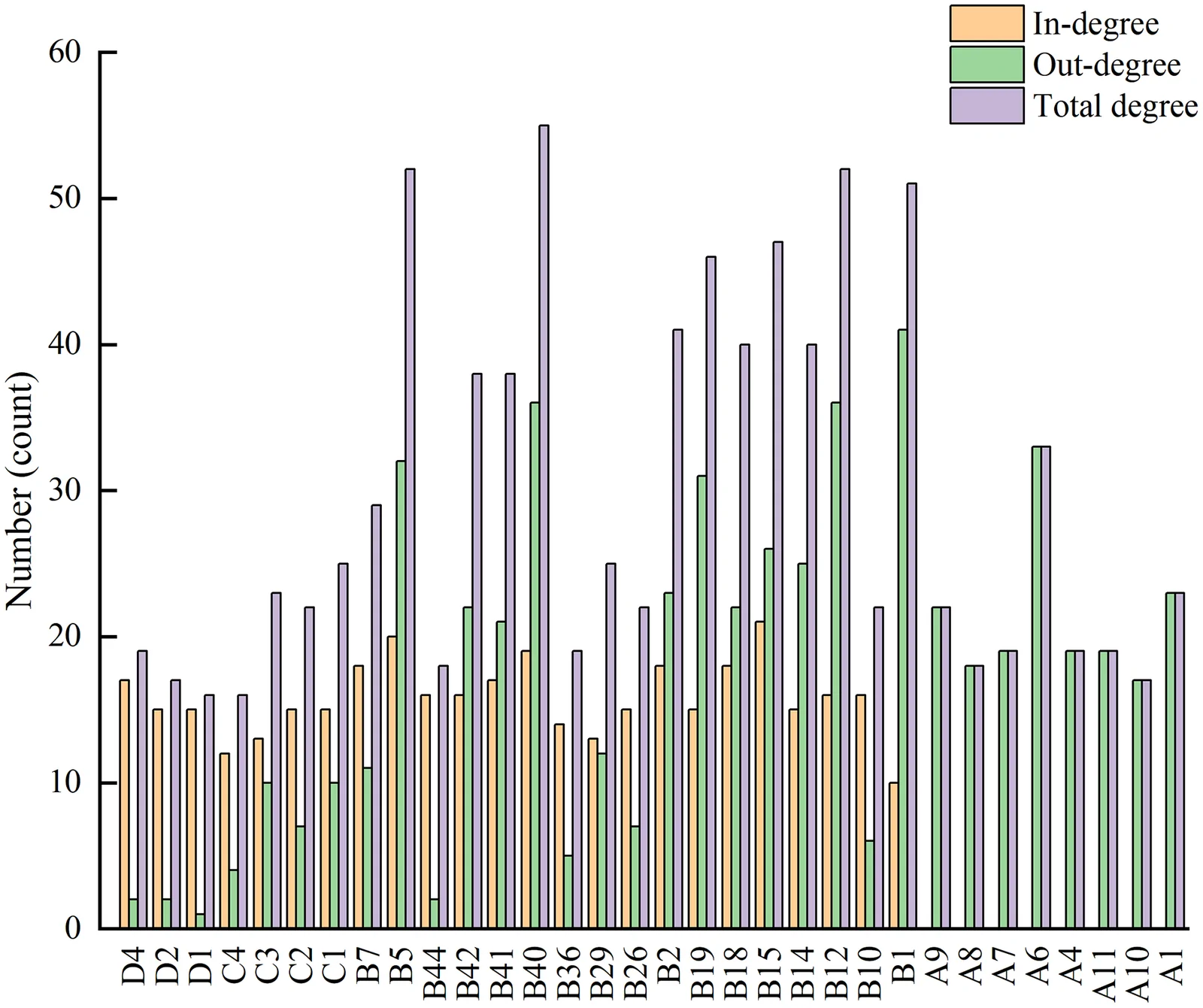
Nodes whose total degree is greater than the average degree and their degree distribution. For each selected node, the bars show the in-degree, out-degree, and total degree calculated from the directed risk-factor network shown in Fig 4. In-degree denotes the number of incoming edges, out-degree denotes the number of outgoing edges, and total degree is the sum of the two.

In the subway operational accident risk factor network, highly connected nodes typically represent core factors that are closely associated with numerous other risk factors, indicating that they may have a broad influence within the risk system. Fig 5 presents the nodes with total degrees greater than the average degree, along with their degree distribution. The node with the highest total degree is B40 (Insufficient safety oversight of subcontractors), followed by B12 (Lack of targeted content in safety training) and B5 (Inadequate enforcement of safety supervision and inspection systems). This indicates that these risk factors are causally linked to most other risk factors in the network. Risk factors with high in-degree values include B15 (Inadequate identification of safety risks), B5, B40, B7 (Failure to follow construction operation procedures), B2 (Failure to effectively implement the all-staff safety responsibility system), and B18 (Blind spots in hidden danger identification). These nodes directly receive interactive information transmitted from most other risk factors in the network, making them highly susceptible to the influence of multiple causes. Risk factors with high out-degree values include B1 (Unclear safety responsibilities for employees), B40, and B12, suggesting that these nodes can directly transmit interactive information to a large number of other nodes, thereby influencing a wide range of risk factors.

**Fig 6:**
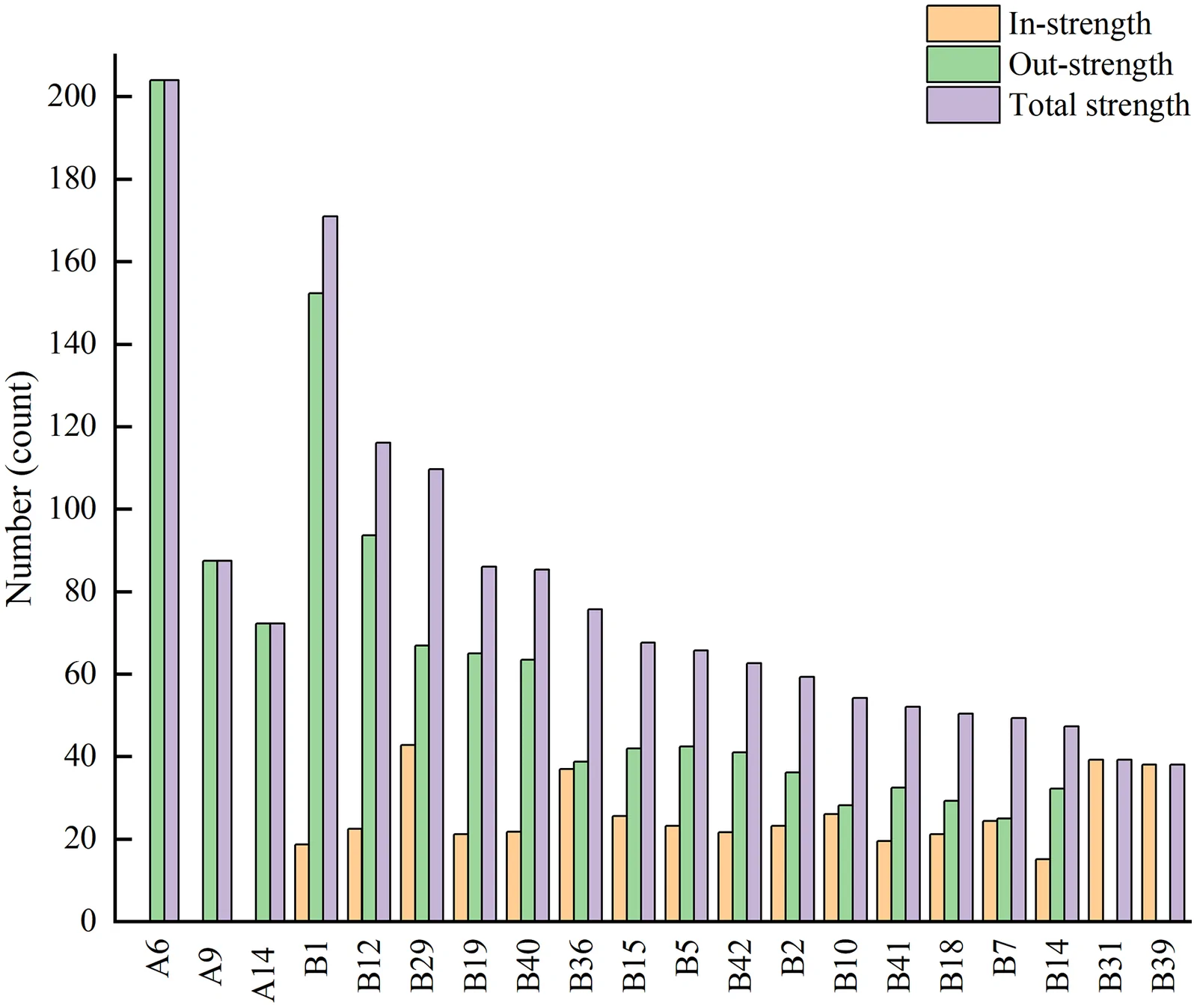
Nodes whose total strength value is greater than the average strength value and their strength distribution. For each selected node, the bars show the in-strength, out-strength, and total strength calculated from the directed weighted network shown in Fig 4. In-strength and out-strength are the sums of the weights of incoming and outgoing edges, respectively, and total strength is the sum of the two.

Node strength refers to the sum of the weights of all edges connected to a given node. Nodes with higher strength values are connected to more nodes and are more likely to contribute to the occurrence of accidents. Fig 6 displays the nodes with total strength values greater than the average, along with their respective strength distributions. It can be observed that the overall distribution trends of node degree and node strength are generally similar, though discrepancies may exist for certain nodes. The node with the highest total strength is A6 (Safety responsibility of managers), followed by B1 (Unclear safety responsibilities for employees) and B12. These findings suggest that these risk factors play a pivotal role in the subway operation accident risk factor network.

Mutual information, a key metric in probability theory, is used to quantify the strength of dependence between two random variables. In subway operation accidents, the relationships between risk factors generally exhibit nonlinear coupling characteristics, and mutual information can effectively quantify these complex interactions. Therefore, in the constructed subway operational accident risk factor network, mutual information can effectively identify key nodes that are strongly associated with numerous other nodes, offering deeper insights into the complex interactions between risk factors than the betweenness centrality, which only identifies the shortest paths. Moreover, mutual information has been applied to accident risk analysis in fields such as railway transportation [58] and maritime operations [59]. Based on mutual information theory, the information content of each node was calculated, and the nodes were ranked accordingly. The top 10 nodes are listed in Table 6. As shown in the table, 8 of these key nodes are related to management factors, indicating the significant role of management in the subway operation accident network. Implementing preventive and control measures targeting safety management factors such as B1 (Unclear safety responsibilities for employees), B40 (Insufficient safety oversight of subcontractors), B12 (Lack of targeted content in safety training), and B5 (Inadequate enforcement of safety supervision and inspection systems) can effectively slow down risk evolution and significantly reduce the incidence of subway operation accidents.

**Table 6:** Top 10 subway operation accident risk factors ranked by the mutual-information-based node importance score.

No.	Node	information content	No.	Node	information content
1	B1	305.52	6	A9	79.45
2	A6	259.65	7	B5	51.77
3	B12	156.25	8	B29	30.20
4	B40	108.38	9	B15	29.01
5	B19	80	10	B42	28.45

**Note:** The node importance score I(i) was calculated as defined in Section 2.5.5 and Eqs. (14)–(15). A higher I(i) indicates a greater relative information contribution and systemic importance of the corresponding risk factor in the directed weighted risk-factor network. Risk-factor codes and their definitions are provided in Table 2.

To verify the effectiveness of key factor identification, this study compared the key factors identified using the mutual information method with those identified using traditional network topology metrics. The results are presented in Table 7. A high degree of consistency was observed among the top ten nodes identified by the three methods. Specifically, six nodes, namely B1, B12, B40, B5, B15, and B19, appeared in the top ten rankings across all three methods. This result not only demonstrates the importance of key risk factors such as B1, B12, and B40, but also indirectly supports the validity of the key factor identification approach adopted in this study. In addition, the mutual information method uniquely included two safety culture risk factors, namely A6 and A9, in the top ten rankings, whereas these nodes ranked only 12th and 17th in degree centrality analysis, respectively. Moreover, their betweenness centrality values were zero because they possessed only outgoing edges. This discrepancy highlights the advantage of the mutual information method. Within the framework of the 24Model, safety culture is explicitly defined as the highest-level organizational factor in accident causation. It directly affects the effectiveness of the safety management system and indirectly influences individual behaviors and conditions through managerial mechanisms. Previous studies have shown that, in accident cases analyzed using the 24Model, defects in organizational safety culture are often accompanied by systematic defects in the safety management system, and together they constitute the underlying organizational causes of accidents [58]. However, such root-level factors are easily overlooked in traditional accident risk analysis.

**Table 7:** Comparison of the top 10 subway operation accident risk-factor rankings obtained using mutual-information-based node importance, node degree, and betweenness centrality.

No.	Mutual information	Degree	Betweenness centrality
1	B1	B40	B12
2	A6	B12	B1
3	B12	B5	B40
4	B40	B1	B5
5	B19	B15	B29
6	A9	B19	B15
7	B5	B2	B36
8	B29	B18	B19
9	B15	B14	B18
10	B42	B42	B10

**Note:** The table compares risk-factor rankings obtained using three node-importance measures: mutual-information-based node importance, node degree, and betweenness centrality. Mutual-information-based node importance is defined in Section 2.5.5 and Eqs. (14)–(15); node degree is defined in Section 2.5.2 and Eqs (6)–(8); and betweenness centrality is defined in Section 2.5.4 and Eqs. (12)–(13). These measures characterize complementary aspects of the structural or systemic importance of risk factors in the constructed complex network. Risk-factor codes and their definitions are provided in Table 2.

Although A6 and A9 appeared relatively infrequently in accident reports, the corresponding association rules exhibited high lift values, indicating that once these factors emerged, they tended to produce strong coupling effects with other safety management system defects. Traditional topological metrics have difficulty capturing such deep-rooted risk factors characterized by low occurrence frequency but high impact. In contrast, the mutual information method can effectively identify these fundamental safety culture factors and compensate for the limitations of insufficient organizational-level risk factor mining. Therefore, the mutual information method can provide a more comprehensive perspective for identifying key risk factors.

### 3.5. Robustness analysis

Network robustness refers to the ability of a network to maintain its structural stability when certain nodes or edges fail. Disrupting the interaction network of risk factors in subway operations and reducing its stability can, to some extent, prevent the evolution of accident risks. In this study, node-targeted attack strategies are employed to analyze changes in network robustness, aiming to provide decision-making support for reducing the occurrence of subway operation accidents.

**Fig 7:**
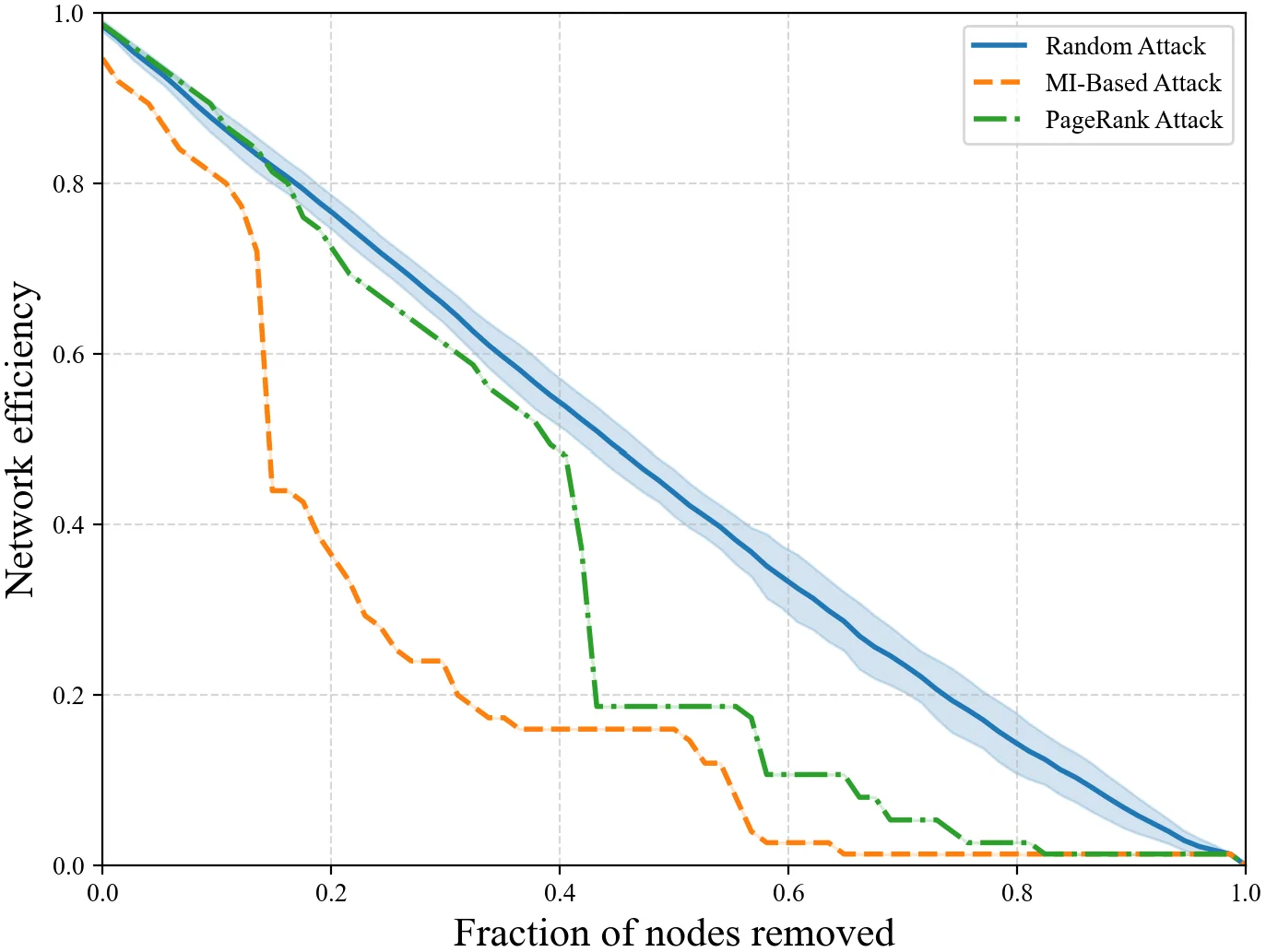
Network robustness analysis results. Starting from the network shown in Fig 4, nodes were sequentially removed using one random strategy based on Monte Carlo simulation and two targeted strategies based on descending mutual information and PageRank rankings. Network efficiency was recalculated after each node-removal step and plotted against the fraction of nodes removed. The solid blue line represents the mean result of 50 random simulations, and the shaded area represents mean ± standard deviation.

Two attack strategies—random attacks based on Monte Carlo simulation and targeted attacks based on node importance ranking—were employed to simulate the removal of network nodes and observe changes in network robustness, as shown in Fig 7. The number of iterations in the Monte Carlo simulation was set to 50. Under the random attack strategy, each simulation adopted a randomly generated node removal sequence, and a fixed random seed (42) was used to ensure reproducibility. For the deliberate attack strategies based on mutual information and PageRank, nodes were removed in descending order of importance. In each simulation, the size of the largest connected component of the remaining network was recorded at the current node removal ratio, after which the mean value and standard deviation across the 50 simulations were calculated. As illustrated, under both random and targeted attacks, the robustness of the network decreases progressively as the number of failed nodes increases. Comparison results show that targeted attacks lead to a faster degradation of network robustness than random attacks, indicating that proactively controlling key risk factors is more effective in preventing subway operation accidents. Moreover, compared to targeted attacks based on the PageRank algorithm, those based on the mutual information theory cause the network to collapse more rapidly. This suggests that the node importance ranking method based on mutual information theory is more suitable for identifying critical risk factors in the subway accident risk factor network.

## 4. Discussion

This study explores the application of the 24Model, association rule mining, and complex network in analyzing the risk factors of subway operation accidents. A risk factor network model of subway operation accidents was constructed, through which the key risk factors were identified. The findings provide theoretical support for preventing the escalation of risks in subway operations and for formulating effective accident prevention strategies.

The analysis results indicate that safety management factors are the primary risk factors in subway operation accidents. Among them, unclear safety responsibilities of employees (B1 = 305.52), lack of targeted content in safety training (B12 = 156.25), and insufficient safety oversight of subcontractors (B40 = 108.38) have significant impacts on accident occurrence and are identified as the most critical risk factors. Previous studies on risk factors in subway operation accidents have primarily focused on human, equipment, and environmental dimensions. This difference may arise from the analytical scope and data representation adopted in previous studies. Methods based primarily on fault trees, predefined assessment indices, or surface-level text frequencies tend to emphasize directly observable failures, such as human errors and equipment malfunctions. In contrast, the 24Model used in this study enables immediate failures to be traced back to defects in safety capabilities, safety management systems, and safety culture. The network-based analysis further captures the repeated connections of these organizational factors with multiple accident scenarios, which may explain their higher importance in the present results. For instance, Wang et al. [4] analyzed 910 subway operation accidents and identified key issues and corresponding control measures from the perspectives of unsafe human behavior and equipment. Liu et al. [60] developed an accident prediction model based on convolutional neural networks and fault tree analysis, which can forecast accident consequences using factors such as the number of stations on lines, headways of lines, and the total operating months of a line. He et al. [20] constructed a risk assessment index system from the dimensions of personnel, equipment, and environment using fault tree analysis and identified vehicle system failures and signal system failures as the most critical risk factors through the AHP method. As a complex system, subway operation involves accidents that often result from the coupling of multiple risk factors related to humans, equipment, safety management, and the environment. Therefore, this study supplements the understanding of the relationship between safety management risk factors and accidents under specific scenarios (i.e., subway operation), thus further expanding the research scope on risk factors in subway operation accidents. Most existing studies rely on a single approach, such as statistical investigation or accident analysis, while relatively few have adopted an integrated methodological framework. Wang et al. [5] identified key risk factors based on 910 accidents using semantic network analysis and word frequency statistics, with a primary focus on surface-level information extraction rather than the construction of deep accident causation networks. He et al. [20] employed fault tree analysis and data envelopment analysis to identify risk factors, mainly evaluating risks from the three dimensions of human, equipment, and environment, but without sufficiently revealing the coupling and transmission relationships among factors. By integrating the 24Model, association rule mining, and complex network theory, the present study systematically analyzed accident risk factors across four stages at both organizational and individual levels, thereby extending, to a certain extent, the methodological research on key factor analysis in subway operation accidents. Furthermore, in the academic application of the 24Model, existing studies applying the 24Model have often focused on immediate causes, such as unsafe acts and unsafe conditions, as well as safety management system defects, whereas quantitative research on safety culture defects remains relatively limited. Through association rule mining and complex network methods, this study identified safety culture defect factors, such as A6 and A9, as occupying central positions within the subway operation safety risk network, and successfully verified their critical root-cause roles in accident causation.

Compared with existing studies, the unique contributions of this study are reflected in the following aspects. First, previous studies employing similar methods have mainly focused on fields such as coal mining [35], chemical engineering [30], and highways [48], whereas the present study specifically concentrates on subway operation accidents. Subway operation systems possess distinctive characteristics, including high-density passenger flow, enclosed spaces, and multi-system coupling. Consequently, their risk factor systems differ substantially from those in coal mining, chemical engineering, and related fields. Moreover, data-driven studies on key risk factor analysis in subway operation safety remain relatively limited. This study further extends the application of the integrated framework combining the 24Model, association rule mining, and complex network theory in the field of subway operation safety. Second, regarding the application of the 24Model, existing studies have primarily focused on individual unsafe acts, unsafe conditions, or safety management system defects [43], while quantitative analysis of safety culture defect factors has remained relatively insufficient. Few previous studies have quantitatively incorporated safety-culture factors within the 24Model into a complex-network framework. This study addresses this gap by systematically integrating these factors into the network analysis and identifying A6 (safety responsibility of managers) and A9 (responsibilities for safety in every department) as prominent factors within the safety-culture category. To the best of our knowledge, this finding has not previously been reported in the field of subway operation safety, thereby providing quantitative validation support for the analysis of safety culture factors within the 24Model framework. Third, although association rule mining and complex network methods have previously been applied in accident risk analysis, this study is the first to organically integrate the 24Model, association rule mining, complex network theory, and mutual information theory into a complete analytical framework encompassing factor identification, rule mining, network modeling, and importance ranking. This integrated methodology, combining theory-driven analysis, data-driven mining, topological analysis, and information-based importance ranking, provides a distinctive methodological advantage for the present study. From a network perspective, the high importance of B1, B12, and B40 indicates that these factors are not merely frequent causes in individual accidents, but are repeatedly connected with multiple downstream unsafe acts, unsafe conditions, and other management defects. Their high degree, strength, or information values suggest that changes in these factors may affect a relatively large portion of the accident-causation network. Similarly, the central positions of A6 and A9 indicate that safety-culture defects may function as upstream organizational conditions that influence multiple management and behavioral factors rather than acting as isolated causes.

The key risk factors identified in this study have practical implications not only for the internal safety management of subway operating organizations but also for regulatory oversight and policy implementation in urban rail transit. China’s current regulatory framework for urban rail transit safety emphasizes the primary responsibility of operating organizations for graded risk control and hidden-hazard identification and rectification, with safety responsibilities required to be progressively assigned to specific departments and positions. Accordingly, the key factors identified in this study, such as unclear safety responsibilities of employees and safety responsibility of managers, are closely aligned with current policy requirements concerning the implementation of position-specific and managerial safety responsibilities. For regulators, highly important risk factors identified through complex network analysis can provide supplementary evidence for prioritizing supervision and safety assessment, allowing limited regulatory resources to focus on organizational and managerial factors with strong network connectivity and potential cascading influence rather than only on direct risks such as equipment failures. In addition, the identification of insufficient safety oversight of subcontractors and lack of targeted content in safety training suggests that regulatory inspections and third-party safety assessments could place greater emphasis on whole-process contractor safety management, position-specific training, and the effective implementation of related management measures. These applications are consistent with the principles of responsibility implementation, dynamic risk management, and closed-loop hazard governance emphasized in the current regulatory framework for urban rail transit operation safety. Therefore, the proposed approach may serve as a supplementary analytical tool within existing regulatory frameworks by identifying systemically important risk factors from accident data and providing evidence to support the prioritization of preventive safety management measures by regulators and operating organizations.

The risk factor with the highest information value is the unclear safety responsibilities of employees. For example, in a major subway flooding accident, although the emergency plan was activated, unclear responsibilities for flood control command on-site and the lack of unified dispatching led to delayed responses and chaotic rescue operations. Similarly, in a metro rear-end collision, the dispatcher, under special snowy conditions, had an unclear understanding of their safety responsibilities regarding train coupling and decoupling commands, leading to improper operations that became a critical link in the accident chain. Therefore, enhancing employees’ understanding of their own job-specific safety responsibilities and ensuring proper fulfillment of these responsibilities can help prevent subway operation accidents. Enterprises can improve internal safety management systems and intensify efforts to promote awareness of job-specific safety responsibilities, thereby preventing the situation where a small number of employees remain unclear about their own duties. Subway operation enterprises may establish a “one position, one responsibility, one checklist” system by developing position-specific safety responsibility checklist cards for dispatchers, drivers, station attendants, maintenance personnel, and other employees. These cards should explicitly specify required responsibilities, prohibited actions, reporting channels for hidden dangers, and reporting time limits. By implementing comprehensive safety responsibility checklists and duty performance assessment checklists for all personnel, safety management indicators can be quantitatively refined, thereby decomposing safety responsibilities to every position and individual employee.

Compared to in-house employees, outsourced workers are generally associated with a higher probability of accidents. Thus, strengthening safety oversight of subcontractors is essential to ensuring the overall safety of enterprises [20]. Enterprises can establish and improve management systems for outsourced units by incorporating external construction teams into the company’s safety management framework. This includes creating detailed personnel files and management records, conducting joint pre-job training with in-house employees, and providing targeted safety awareness programs based on job characteristics to ensure synchronized management of both internal and external personnel. A safety credit record can also be established for outsourced units, not only reviewing their qualifications but also using performance data such as historical accident rates and hazard rectification rates as core indicators for admission and contract renewal. Subway operation enterprises may also establish a “red-yellow-green” access management mechanism for subcontractors. Under this mechanism, subcontractors assigned a green code (no safety accidents within one year and a consistently high hazard-rectification rate defined according to the operator’s regulatory and contractual requirements) may normally undertake operational tasks; those assigned a yellow code (having experienced minor accidents or with a rectification rate below 95%) are required to submit rectification reports and pass reinspection before undertaking new projects; and those assigned a red code (having experienced major or more severe accidents, or committed serious violations) should be immediately removed and placed on a blacklist. Meanwhile, process supervision should be further strengthened by conducting monthly performance assessments of subcontractors. The assessment indicators may include core metrics such as safety training coverage rate, hidden danger identification and rectification rate, and accident occurrence rate. The assessment results should serve as a direct basis for contract renewal and settlement decisions.

Safety training is an essential means of preventing unsafe behaviors. However, with the expansion of operational scale and the changing characteristics of employees, traditional safety training methods are no longer adequate to meet the targeted safety training needs for accident prevention [61]. Therefore, enterprises can leverage accident case data as a basis, compare accident causes with industry standards and regulations, and tailor training content based on the company’s specific context. Supplementary training materials such as PPT presentations, manuals, and videos can be developed to enhance the specificity and effectiveness of safety training programs [62]. Subway operation enterprises may establish a “hierarchical and position-specific” training content system. For example, the integrated training model of “real-name safety codes + VR warning education + multimedia instruction” may be introduced to transform safety knowledge into immersive learning content through information technology. Training courses can then be tailored according to the risk characteristics of different positions, while implementing the management approach of “one person, one code, one file” to enhance the specificity and traceability of safety training. Meanwhile, employee safety training records may be established within a clearly specified period after completion of pre-job safety education after the completion of the three-level pre-job safety education program, thereby ensuring that training records are accessible and assessment results are fully traceable.

As shown in Table 6, among safety culture-related risk factors, the “Safety responsibility of managers” is identified as a major risk factor influencing subway operation accidents. This finding is consistent with existing studies. For example, Tappura’s research highlights that the safety responsibility of managers is a key component of effective safety management, culture, and climate [63]. Meanwhile, this finding is also consistent with the global trend in metro safety management practices. Internationally recognized safety management systems, such as ISO 45001, likewise emphasize leadership commitment and full employee participation, regarding managerial safety commitment as the foundation for the effective operation of the safety management system. Therefore, metro operation enterprises may establish a “dual assessment” mechanism for managerial safety performance, directly linking safety performance with bonus allocation and position promotion. For example, enterprises may sign “safety production responsibility agreements” that clearly specify annual safety management objectives and corresponding economic assessment requirements. At the same time, a bidirectional evaluation mechanism involving both department heads and supervisory leaders may be implemented, with assessment results further extended to cooperative teams, thereby promoting the cascading implementation of safety responsibilities through reward and penalty mechanisms. In addition, the enterprise safety director may organize monthly inspections of managerial safety performance. The inspection contents may include whether managers conduct team-led safety inspections as required, approve hidden danger rectification within the specified time limits, and participate in emergency drills. All inspection results should be documented and archived in written form.

In this study, an exploratory stratified analysis was conducted using train collision accidents, which constituted the largest accident category in the dataset. The key risk factors identified from this subgroup were compared with those obtained in Section 3.4 to examine the robustness of the identified key risk factors across different accident scenarios. The results showed that risk factors such as unclear safety responsibilities for employees, inadequate enforcement of safety supervision and inspection systems, and failure to conduct regular emergency drills still exhibited high importance in train collision accidents. This indicates that these safety management system defects represent common factors across different accident types, thereby further supporting the robustness of the key risk factors identified in this study. Meanwhile, the results also revealed that factors such as insufficient safety oversight of subcontractors, failure to promptly and effectively rectify hidden dangers, and inadequate identification of safety risks exhibited reduced importance in train collision accidents, reflecting the specificity of accident types. Train collision accidents are often directly associated with train dispatching, signaling systems, and on-site operational safety. Consequently, factors directly related to operational procedures demonstrated greater importance in this accident category, including failure to implement shift handover procedures, failure to inform employees of existing safety risks and countermeasures, failure to effectively implement the all-staff safety responsibility system, and failure to follow construction operation procedures. These findings indicate that different accident types indeed exhibit variations in risk coupling patterns, while certain key risk factors still demonstrate robustness across accident categories. However, due to the limited sample size for individual accident types, it was not feasible to conduct effective stratified analyses for each category separately. Therefore, future research may further expand the accident case database and carry out more systematic stratified analyses covering all accident types.

## 5. Conclusions

This study analyzed 76 subway operation accidents that occurred across 16 cities in China between 2009 and 2024, covering 13 categories of operational accidents, including train collisions, train derailments, train conflicts, and flooding and backflow intrusion in stations and track areas. The geographical and temporal scope was determined by considering the comparability of accident data, the development trajectory of China’s urban rail transit, and the completeness of accident information. The period from 2009 to 2024 covers an important stage in which China’s urban rail transit developed from rapid system expansion toward large-scale networked operation, while restricting the study to China helps maintain the comparability of cases under relatively consistent accident-reporting practices, regulatory frameworks, and accident classification standards. In addition, priority was given to cases containing sufficiently detailed information on accident processes and causation to support in-depth coding using the 24Model, thereby improving the reliability of risk-factor extraction. Based on these accident data, this study integrated the 24Model, Apriori association rule mining, complex network analysis, and mutual information theory to identify risk factors, characterize their associations, and determine systemically important factors in subway operation accidents. The results identified unclear safety responsibilities of employees, safety responsibility of managers, insufficient safety oversight of subcontractors, and lack of targeted content in safety training as the four most critical risk factors. These findings provide evidence to support the prioritization of risk control and the development of targeted accident-prevention measures in subway operations.

This study collected only 76 subway operational accident cases, which still constitutes a relatively small sample size, leading to certain limitations in the scope and quantity of the case database. The small sample size, when combined with the Apriori algorithm, may affect the generalizability of the research conclusions. Therefore, a limitation of this study is the exploratory construction of a complex network of subway accident risk factors under strict constraints, focusing on the analysis of key risk factors. In addition, the accident case database consists of incidents that have already resulted in severe consequences. Although this data collection strategy ensures the authenticity and authority of the cases, it inevitably introduces limitations in data sources, as Near Misses data are lacking, which may affect the representativeness of the research findings. In addition, due to differences in individual knowledge backgrounds, experts may interpret the same accident text differently, particularly with respect to latent risk factors such as safety culture defects and safety management system defects. It should be noted that expert judgment is a commonly adopted approach in this type of research and possesses irreplaceable value in safety analysis. Nevertheless, appropriate methodological measures are required to minimize potential bias. In the present study, expert knowledge bias was mitigated to a certain extent through measures such as independent multi-expert coding and arbitration mechanisms. Furthermore, accident investigation reports from different years and sources may vary in terms of detail and investigation depth. As a result, textual descriptions related to organizational-level accident causes may be insufficiently detailed, thereby limiting the in-depth exploration of organizational causation factors. This limitation is also a common issue in studies based on accident investigation report data. To address this problem, the present study only extracted factors supported by explicit textual evidence or directly inferable information, while adhering to the principle of excluding uncertain factors during the coding process, thereby reducing the influence of report text quality bias to some extent. These limitations indicate that the findings of this study should be regarded as exploratory results derived within a specific sample and methodological framework.

In future studies on subway operation accidents, efforts will be made to increase the number of accident cases and contributing factors, in order to construct a more comprehensive and accurate complex network model. Future research will also incorporate near-miss event data to enrich the diversity of sample types and statistical variability, thereby further enhancing the generalizability of the model and the universality of the research findings. Furthermore, safety risk assessment research will be carried out to provide decision-making support for accident prevention.

## Supporting information

S1 File Association rules of risk factors. (DOCX)

S2 File Results of node degree and node strength of risk factors. (DOCX)

S3 File Mean and standard deviation of the giant connected component (GCC) size under three attack strategies at varying node removal fractions. (DOCX)

S4 File Structured accident case dataset. (XLSX)
