# Supplementary material for: Comprehensive analysis method of key risk factors in the subway operation accident by complex network and accident data
Source: PLoS One. 2026 Sep 18;21(9):e0358549. doi: 10.1371/journal.pone.0358549 (PMC13588349; doi:10.1371/journal.pone.0358549)
Supplement: Supplementary file 1 [file pone.0358549.s001.docx]

**Supplementary file 1**

## **Association rules of risk factors**

| **Post item** | **Front item** | **Support** | **Confidence** | **Lift** |
| --- | --- | --- | --- | --- |
| Construction workers illegal operation | Failure to follow construction operation procedures | 0.26 | 0.91 | 2.03 |
| Insufficient safety consciousness | Inadequate enforcement of safety supervision and inspection systems | 0.45 | 0.88 | 1.29 |
| Failure to follow construction operation procedures | Insufficient supervision of on-site operations | 0.18 | 0.86 | 2.96 |
| Construction workers illegal operation | Insufficient supervision of on-site operations | 0.18 | 0.86 | 1.92 |
| Insufficient safety consciousness | Failure to effectively implement the all-staff safety responsibility system | 0.34 | 0.85 | 1.24 |
| Failure to effectively implement the all-staff safety responsibility system | Failure to promptly and effectively rectify identified hidden danger | 0.16 | 0.83 | 2.44 |
| Insufficient security knowledge | Lack of targeted content in safety training | 0.16 | 0.83 | 2.64 |
| Construction workers illegal operation | Inadequate on-site safety inspection | 0.29 | 0.73 | 1.63 |
| Insufficient safety consciousness | Inadequate on-site safety inspection | 0.29 | 0.73 | 1.06 |
| Insufficient safety consciousness | Inadequate identification of safety risks | 0.29 | 0.73 | 1.06 |
| Inadequate enforcement of safety supervision and inspection systems | Failure to effectively implement the all-staff safety responsibility system | 0.34 | 0.69 | 1.55 |
| Construction workers illegal operation | Failure to promptly and effectively rectify identified hidden danger | 0.16 | 0.67 | 1.49 |
| risk-taking mentality | Failure to promptly and effectively rectify identified hidden danger | 0.16 | 0.67 | 2.30 |
| Inadequate enforcement of safety supervision and inspection systems | Failure to promptly and effectively rectify identified hidden danger | 0.16 | 0.67 | 1.49 |
| Ineffective safety training outcomes | Inadequate enforcement of safety supervision and inspection systems | 0.45 | 0.65 | 1.54 |
| Inadequate enforcement of safety supervision and inspection systems | Workplace audits and inspections | 0.45 | 0.65 | 1.45 |
| Inadequate maintenance of equipment and facilities | Facility satisfaction | 0.37 | 0.64 | 2.71 |
| Blind spots in hidden danger identification | Inadequate identification of safety risks | 0.29 | 0.64 | 1.86 |
| Failure to follow construction operation procedures | Inadequate on-site safety inspection | 0.29 | 0.64 | 2.20 |
| Construction workers illegal operation | risk-taking mentality | 0.29 | 0.64 | 1.42 |
| Inadequate enforcement of safety supervision and inspection systems | Inadequate on-site safety inspection | 0.29 | 0.64 | 1.42 |
| Construction workers illegal operation | Failure to effectively implement the all-staff safety responsibility system | 0.34 | 0.62 | 1.38 |
| Insufficient safety consciousness | Blind spots in hidden danger identification | 0.34 | 0.62 | 0.90 |
| Blind spots in hidden danger identification | Workplace audits and inspections | 0.45 | 0.59 | 1.72 |
| Failure to effectively implement the all-staff safety responsibility system | Workplace audits and inspections | 0.45 | 0.59 | 1.72 |
| Inadequate on-site safety inspection | Safety of contractors and subsidiaries | 0.50 | 0.58 | 2.00 |
| Inadequate enforcement of safety supervision and inspection systems | Safety of contractors and subsidiaries | 0.50 | 0.58 | 1.29 |
| Construction workers illegal operation | Insufficient safety consciousness | 0.68 | 0.58 | 1.29 |
| Inadequate enforcement of safety supervision and inspection systems | Demand of safety training | 0.58 | 0.55 | 1.22 |
| Insufficient supervision of on-site operations | Failure to follow construction operation procedures | 0.29 | 0.55 | 2.96 |
| Failure to effectively implement the all-staff safety responsibility system | Inadequate on-site safety inspection | 0.29 | 0.55 | 1.59 |
| Failure to effectively implement the all-staff safety responsibility system | Failure to follow construction operation procedures | 0.29 | 0.55 | 1.59 |
| Failure to effectively implement the all-staff safety responsibility system | Inadequate identification of safety risks | 0.29 | 0.55 | 1.59 |
| Ineffective safety training outcomes | Inadequate on-site safety inspection | 0.29 | 0.55 | 1.30 |
| Inadequate enforcement of safety supervision and inspection systems | Inadequate identification of safety risks | 0.29 | 0.55 | 1.22 |
| Inadequate identification of safety risks | Blind spots in hidden danger identification | 0.34 | 0.54 | 1.86 |
| risk-taking mentality | Failure to effectively implement the all-staff safety responsibility system | 0.34 | 0.54 | 1.86 |
| Ineffective safety training outcomes | Failure to effectively implement the all-staff safety responsibility system | 0.34 | 0.54 | 1.28 |
| Failure to effectively implement the all-staff safety responsibility system | Inadequate enforcement of safety supervision and inspection systems | 0.45 | 0.53 | 1.55 |
| Failure to effectively implement the all-staff safety responsibility system | Individual involvement in safety | 0.45 | 0.53 | 1.55 |
| Construction workers illegal operation | Inadequate enforcement of safety supervision and inspection systems | 0.45 | 0.53 | 1.18 |
| Inadequate enforcement of safety supervision and inspection systems | Individual involvement in safety | 0.45 | 0.53 | 1.18 |
| Insufficient safety oversight of subcontractors | Safety of contractors and subsidiaries | 0.50 | 0.53 | 2.00 |
| Inadequate enforcement of safety supervision and inspection systems | Safety consciousness | 0.71 | 0.52 | 1.16 |
| Inadequate enforcement of safety supervision and inspection systems | Safety importance | 0.76 | 0.48 | 1.08 |
| Failure to follow construction operation procedures | Safety of contractors and subsidiaries | 0.50 | 0.47 | 1.64 |
| Ineffective safety training outcomes | Workplace audits and inspections | 0.45 | 0.47 | 1.12 |
| Inadequate on-site safety inspection | Failure to effectively implement the all-staff safety responsibility system | 0.34 | 0.46 | 1.59 |
| Failure to follow construction operation procedures | Failure to effectively implement the all-staff safety responsibility system | 0.34 | 0.46 | 1.59 |
| Inadequate identification of safety risks | Failure to effectively implement the all-staff safety responsibility system | 0.34 | 0.46 | 1.59 |
| Inadequate maintenance of equipment and facilities | Blind spots in hidden danger identification | 0.34 | 0.46 | 1.95 |
| Inadequate enforcement of safety supervision and inspection systems | Blind spots in hidden danger identification | 0.34 | 0.46 | 1.03 |
| Failure to complete approval procedures | Failure to follow construction operation procedures | 0.29 | 0.45 | 2.88 |
| Construction workers illegal operation | Inadequate identification of safety risks | 0.29 | 0.45 | 1.02 |
| risk-taking mentality | Inadequate on-site safety inspection | 0.29 | 0.45 | 1.57 |
| Ineffective safety training outcomes | Inadequate identification of safety risks | 0.29 | 0.45 | 1.08 |
| Ineffective safety training outcomes | Safety consciousness | 0.71 | 0.44 | 1.06 |
| Power supply systems failures | Inadequate maintenance of equipment and facilities | 0.24 | 0.44 | 2.81 |
| Insufficient security knowledge | Luck-driven mentality | 0.37 | 0.43 | 1.36 |
| Failure to effectively implement the all-staff safety responsibility system | Insufficient supervision of on-site operations | 0.18 | 0.43 | 1.25 |
| Luck-driven mentality | Insufficient supervision of on-site operations | 0.18 | 0.43 | 1.16 |
| Dispatchers illegal operation | Drivers illegal operation | 0.18 | 0.43 | 3.26 |
| Dispatchers illegal operation | Insufficient security knowledge | 0.32 | 0.42 | 3.17 |
| Inadequate on-site safety inspection | Inadequate enforcement of safety supervision and inspection systems | 0.45 | 0.41 | 1.42 |
| Failure to follow construction operation procedures | Individual involvement in safety | 0.45 | 0.41 | 1.42 |
| Inadequate identification of safety risks | Workplace audits and inspections | 0.45 | 0.41 | 1.42 |
| risk-taking mentality | Inadequate enforcement of safety supervision and inspection systems | 0.45 | 0.41 | 1.42 |
| Inadequate identification of safety risks | Demand of safety training | 0.58 | 0.41 | 1.41 |
| risk-taking mentality | Insufficient safety consciousness | 0.68 | 0.38 | 1.33 |
| Luck-driven mentality | Insufficient safety consciousness | 0.68 | 0.38 | 1.04 |
| Blind spots in hidden danger identification | Failure to effectively implement the all-staff safety responsibility system | 0.34 | 0.38 | 1.12 |
| Failure to effectively implement the all-staff safety responsibility system | Blind spots in hidden danger identification | 0.34 | 0.38 | 1.12 |
| Failure to complete approval procedures | Failure to effectively implement the all-staff safety responsibility system | 0.34 | 0.38 | 2.44 |
| Failure to promptly and effectively rectify identified hidden danger | Failure to effectively implement the all-staff safety responsibility system | 0.34 | 0.38 | 2.44 |
| Luck-driven mentality | Blind spots in hidden danger identification | 0.34 | 0.38 | 1.04 |
| Failure to effectively implement the all-staff safety responsibility system | Safety importance | 0.76 | 0.38 | 1.11 |
| Blind spots in hidden danger identification | Safety consciousness | 0.71 | 0.37 | 1.08 |
| Inadequate identification of safety risks | Safety consciousness | 0.71 | 0.37 | 1.28 |
| Failure to effectively implement the all-staff safety responsibility system | Safety of contractors and subsidiaries | 0.50 | 0.37 | 1.08 |
| Blind spots in hidden danger identification | Demand of safety training | 0.58 | 0.36 | 1.06 |
| Failure to effectively implement the all-staff safety responsibility system | Demand of safety training | 0.58 | 0.36 | 1.06 |
| Failure to follow train operation procedures | Inadequate identification of safety risks | 0.29 | 0.36 | 2.30 |
| Failure to complete approval procedures | Inadequate on-site safety inspection | 0.29 | 0.36 | 2.30 |
| Drivers illegal operation | Inadequate identification of safety risks | 0.29 | 0.36 | 1.97 |
| risk-taking mentality | Inadequate identification of safety risks | 0.29 | 0.36 | 1.26 |
| Luck-driven mentality | Inadequate on-site safety inspection | 0.29 | 0.36 | 0.99 |
| Luck-driven mentality | Inadequate identification of safety risks | 0.29 | 0.36 | 0.99 |
| Insufficient safety oversight of subcontractors | Inadequate on-site safety inspection | 0.29 | 0.36 | 1.38 |
| Insufficient security knowledge | Inadequate on-site safety inspection | 0.29 | 0.36 | 1.15 |
| Insufficient security knowledge | risk-taking mentality | 0.29 | 0.36 | 1.15 |
| Insufficient security knowledge | Inadequate identification of safety risks | 0.29 | 0.36 | 1.15 |
| Inadequate enforcement of safety supervision and inspection systems | Facility satisfaction | 0.37 | 0.36 | 0.80 |
| Blind spots in hidden danger identification | Inadequate enforcement of safety supervision and inspection systems | 0.45 | 0.35 | 1.03 |
| Blind spots in hidden danger identification | Individual involvement in safety | 0.45 | 0.35 | 1.03 |
| Inadequate on-site safety inspection | Workplace audits and inspections | 0.45 | 0.35 | 1.22 |
| Failure to follow construction operation procedures | Workplace audits and inspections | 0.45 | 0.35 | 1.22 |
| Inadequate identification of safety risks | Inadequate enforcement of safety supervision and inspection systems | 0.45 | 0.35 | 1.22 |
| Inadequate identification of safety risks | Individual involvement in safety | 0.45 | 0.35 | 1.22 |
| Inadequate maintenance of equipment and facilities | Workplace audits and inspections | 0.45 | 0.35 | 1.49 |
| Insufficient safety oversight of subcontractors | Inadequate enforcement of safety supervision and inspection systems | 0.45 | 0.35 | 1.34 |
| Insufficient safety oversight of subcontractors | Workplace audits and inspections | 0.45 | 0.35 | 1.34 |
| Inadequate identification of safety risks | Safety importance | 0.76 | 0.34 | 1.19 |
| Ineffective safety training outcomes | Safety importance | 0.76 | 0.34 | 0.82 |
| Failure to follow construction operation procedures | Safety consciousness | 0.71 | 0.33 | 1.15 |
| Failure to effectively implement the all-staff safety responsibility system | Safety consciousness | 0.71 | 0.33 | 0.97 |
| risk-taking mentality | Insufficient security knowledge | 0.32 | 0.33 | 1.15 |
| Civil infrastructure failures | Inadequate maintenance of equipment and facilities | 0.24 | 0.33 | 4.22 |
| Signaling systems failures | Power supply systems failures | 0.16 | 0.33 | 2.53 |
| Signaling systems failures | Lack of targeted content in safety training | 0.16 | 0.33 | 2.53 |
| Inadequate on-site safety inspection | Lack of targeted content in safety training | 0.16 | 0.33 | 1.15 |
| Inadequate on-site safety inspection | Failure to promptly and effectively rectify identified hidden danger | 0.16 | 0.33 | 1.15 |
| Failure to follow train operation procedures | Failure to promptly and effectively rectify identified hidden danger | 0.16 | 0.33 | 2.11 |
| Failure to follow construction operation procedures | Lack of targeted content in safety training | 0.16 | 0.33 | 1.15 |
| Failure to follow construction operation procedures | Failure to promptly and effectively rectify identified hidden danger | 0.16 | 0.33 | 1.15 |
| Lack of safety training | Failure to promptly and effectively rectify identified hidden danger | 0.16 | 0.33 | 4.22 |
| Failure to promptly report accident information | Failure to follow train operation procedures | 0.16 | 0.33 | 3.17 |
| Failure to conduct regular emergency drills | Lack of targeted content in safety training | 0.16 | 0.33 | 3.17 |
| Inadequate identification of safety risks | Lack of targeted content in safety training | 0.16 | 0.33 | 1.15 |
| Drivers illegal operation | Failure to promptly and effectively rectify identified hidden danger | 0.16 | 0.33 | 1.81 |
| Luck-driven mentality | Lack of targeted content in safety training | 0.16 | 0.33 | 0.90 |
| Maintenance personnel illegal operation | Lack of targeted content in safety training | 0.16 | 0.33 | 3.17 |
| Insufficient safety oversight of subcontractors | Failure to promptly and effectively rectify identified hidden danger | 0.16 | 0.33 | 1.27 |
| Dispatchers illegal operation | Lack of targeted content in safety training | 0.16 | 0.33 | 2.53 |
| Inadequate enforcement of safety supervision and inspection systems | Lack of targeted content in safety training | 0.16 | 0.33 | 0.75 |
| Failure to follow construction operation procedures | Demand of safety training | 0.58 | 0.32 | 1.10 |
| Blind spots in hidden danger identification | Safety importance | 0.76 | 0.31 | 0.91 |
| Failure to follow construction operation procedures | Safety importance | 0.76 | 0.31 | 1.07 |
| Drivers illegal operation | Failure to effectively implement the all-staff safety responsibility system | 0.34 | 0.31 | 1.67 |
| Construction workers illegal operation | Blind spots in hidden danger identification | 0.34 | 0.31 | 0.69 |
| Insufficient safety oversight of subcontractors | Failure to effectively implement the all-staff safety responsibility system | 0.34 | 0.31 | 1.17 |
| Insufficient security knowledge | Blind spots in hidden danger identification | 0.34 | 0.31 | 0.97 |
| Inadequate on-site safety inspection | Safety consciousness | 0.71 | 0.30 | 1.02 |
| Unclear safety responsibilities for employees | Individual involvement in safety | 0.45 | 0.29 | 2.24 |
| Insufficient supervision of on-site operations | Individual involvement in safety | 0.45 | 0.29 | 1.60 |
| Inadequate on-site safety inspection | Individual involvement in safety | 0.45 | 0.29 | 1.02 |
| Failure to follow construction operation procedures | Inadequate enforcement of safety supervision and inspection systems | 0.45 | 0.29 | 1.02 |
| Failure to promptly and effectively rectify identified hidden danger | Workplace audits and inspections | 0.45 | 0.29 | 1.86 |
| Drivers illegal operation | Inadequate enforcement of safety supervision and inspection systems | 0.45 | 0.29 | 1.60 |
| Luck-driven mentality | Inadequate enforcement of safety supervision and inspection systems | 0.45 | 0.29 | 0.80 |
| Insufficient safety oversight of subcontractors | Individual involvement in safety | 0.45 | 0.29 | 1.12 |
| Insufficient security knowledge | Inadequate enforcement of safety supervision and inspection systems | 0.45 | 0.29 | 0.93 |
| Ineffective safety training outcomes | Individual involvement in safety | 0.45 | 0.29 | 0.70 |
| Inadequate identification of safety risks | Facility satisfaction | 0.37 | 0.29 | 0.99 |
| Blind spots in hidden danger identification | Insufficient supervision of on-site operations | 0.18 | 0.29 | 0.84 |
| Absence of a feasible construction plan | Insufficient supervision of on-site operations | 0.18 | 0.29 | 3.62 |
| Failure to complete approval procedures | Insufficient supervision of on-site operations | 0.18 | 0.29 | 1.81 |
| Absence of a safety risk management mechanism | Insufficient supervision of on-site operations | 0.18 | 0.29 | 3.62 |
| Failure to carry out technical disclosure | Insufficient supervision of on-site operations | 0.18 | 0.29 | 5.43 |
| risk-taking mentality | Insufficient supervision of on-site operations | 0.18 | 0.29 | 0.99 |
| Insufficient supervision of on-site operations | Enforcement of safety rules | 0.66 | 0.28 | 1.52 |
| Inadequate identification of safety risks | Enforcement of safety rules | 0.66 | 0.28 | 0.97 |
| Inadequate on-site safety inspection | Safety importance | 0.76 | 0.28 | 0.95 |
| Insufficient safety oversight of subcontractors | Safety importance | 0.76 | 0.28 | 1.05 |
| Inadequate on-site safety inspection | Demand of safety training | 0.58 | 0.27 | 0.94 |
| Lack of targeted content in safety training | Demand of safety training | 0.58 | 0.27 | 1.73 |
| Blind spots in hidden danger identification | Inadequate on-site safety inspection | 0.29 | 0.27 | 0.80 |
| Insufficient supervision of on-site operations | Inadequate on-site safety inspection | 0.29 | 0.27 | 1.48 |
| Inadequate on-site safety inspection | Inadequate identification of safety risks | 0.29 | 0.27 | 0.94 |
| Absence of a feasible construction plan | Failure to follow construction operation procedures | 0.29 | 0.27 | 3.45 |
| Failure to set up safety warning signs | Failure to follow construction operation procedures | 0.29 | 0.27 | 2.59 |
| Failure to promptly and effectively rectify identified hidden danger | Inadequate identification of safety risks | 0.29 | 0.27 | 1.73 |
| Inadequate identification of safety risks | Inadequate on-site safety inspection | 0.29 | 0.27 | 0.94 |
| Drivers illegal operation | risk-taking mentality | 0.29 | 0.27 | 1.48 |
| Inadequate maintenance of equipment and facilities | Inadequate identification of safety risks | 0.29 | 0.27 | 1.15 |
| Luck-driven mentality | Failure to follow construction operation procedures | 0.29 | 0.27 | 0.74 |
| Insufficient safety oversight of subcontractors | Inadequate identification of safety risks | 0.29 | 0.27 | 1.04 |
| Inadequate identification of safety risks | Safety of contractors and subsidiaries | 0.50 | 0.26 | 0.91 |
| Insufficient safety oversight of subcontractors | Safety consciousness | 0.71 | 0.26 | 0.99 |
| Insufficient supervision of on-site operations | Inadequate enforcement of safety supervision and inspection systems | 0.45 | 0.24 | 1.28 |
| Insufficient supervision of on-site operations | Workplace audits and inspections | 0.45 | 0.24 | 1.28 |
| Failure to follow train operation procedures | Inadequate enforcement of safety supervision and inspection systems | 0.45 | 0.24 | 1.49 |
| Failure to follow train operation procedures | Individual involvement in safety | 0.45 | 0.24 | 1.49 |
| Failure to complete approval procedures | Inadequate enforcement of safety supervision and inspection systems | 0.45 | 0.24 | 1.49 |
| Failure to complete approval procedures | Workplace audits and inspections | 0.45 | 0.24 | 1.49 |
| Failure to promptly and effectively rectify identified hidden danger | Inadequate enforcement of safety supervision and inspection systems | 0.45 | 0.24 | 1.49 |
| Failure to promptly and effectively rectify identified hidden danger | Individual involvement in safety | 0.45 | 0.24 | 1.49 |
| Inadequate maintenance of equipment and facilities | Inadequate enforcement of safety supervision and inspection systems | 0.45 | 0.24 | 0.99 |
| Drivers illegal operation | Insufficient safety consciousness | 0.68 | 0.23 | 1.25 |
| Insufficient security knowledge | Insufficient safety consciousness | 0.68 | 0.23 | 0.73 |
| Unclear safety responsibilities for employees | Blind spots in hidden danger identification | 0.34 | 0.23 | 1.75 |
| Insufficient supervision of on-site operations | Failure to effectively implement the all-staff safety responsibility system | 0.34 | 0.23 | 1.25 |
| Inadequate on-site safety inspection | Blind spots in hidden danger identification | 0.34 | 0.23 | 0.80 |
| Absence of a feasible construction plan | Failure to effectively implement the all-staff safety responsibility system | 0.34 | 0.23 | 2.92 |
| Failure to follow train operation procedures | Blind spots in hidden danger identification | 0.34 | 0.23 | 1.46 |
| Failure to follow train operation procedures | Failure to effectively implement the all-staff safety responsibility system | 0.34 | 0.23 | 1.46 |
| Failure to follow construction operation procedures | Blind spots in hidden danger identification | 0.34 | 0.23 | 0.80 |
| Civil infrastructure failures | Blind spots in hidden danger identification | 0.34 | 0.23 | 2.92 |
| Drivers illegal operation | Blind spots in hidden danger identification | 0.34 | 0.23 | 1.25 |
| Inadequate maintenance of equipment and facilities | Failure to effectively implement the all-staff safety responsibility system | 0.34 | 0.23 | 0.97 |
| Luck-driven mentality | Failure to effectively implement the all-staff safety responsibility system | 0.34 | 0.23 | 0.63 |
| Insufficient safety oversight of subcontractors | Blind spots in hidden danger identification | 0.34 | 0.23 | 0.88 |
| Insufficient security knowledge | Failure to effectively implement the all-staff safety responsibility system | 0.34 | 0.23 | 0.73 |
| Insufficient supervision of on-site operations | Demand of safety training | 0.58 | 0.23 | 1.23 |
| Inadequate maintenance of equipment and facilities | Demand of safety training | 0.58 | 0.23 | 0.96 |
| Insufficient supervision of on-site operations | Safety consciousness | 0.71 | 0.22 | 1.21 |
| Failure to follow train operation procedures | Safety consciousness | 0.71 | 0.22 | 1.41 |
| Lack of targeted content in safety training | Safety consciousness | 0.71 | 0.22 | 1.41 |
| Signaling systems failures | Inadequate maintenance of equipment and facilities | 0.24 | 0.22 | 1.69 |
| Inadequate flood protection capability of safety equipment and facilities | Inadequate maintenance of equipment and facilities | 0.24 | 0.22 | 4.22 |
| Poor quality control in construction project execution | Inadequate maintenance of equipment and facilities | 0.24 | 0.22 | 2.81 |
| Signaling systems failures | Luck-driven mentality | 0.37 | 0.21 | 1.63 |
| Failure to effectively implement the all-staff safety responsibility system | Facility satisfaction | 0.37 | 0.21 | 0.63 |
| Drainage systems failures | Luck-driven mentality | 0.37 | 0.21 | 2.71 |
| Poor quality control in construction project execution | Facility satisfaction | 0.37 | 0.21 | 2.71 |
| Dispatchers illegal operation | Luck-driven mentality | 0.37 | 0.21 | 1.63 |
| Vehicle systems failures | Luck-driven mentality | 0.37 | 0.21 | 2.71 |
| Failure to complete approval procedures | Safety of contractors and subsidiaries | 0.50 | 0.21 | 1.33 |
| Insufficient supervision of on-site operations | Safety importance | 0.76 | 0.21 | 1.12 |
| Failure to follow train operation procedures | Safety importance | 0.76 | 0.21 | 1.31 |
| Failure to complete approval procedures | Safety importance | 0.76 | 0.21 | 1.31 |
| Failure to promptly and effectively rectify identified hidden danger | Safety importance | 0.76 | 0.21 | 1.31 |
| Unclear safety responsibilities for employees | Safety consciousness | 0.71 | 0.19 | 1.41 |
| Failure to promptly and effectively rectify identified hidden danger | Safety consciousness | 0.71 | 0.19 | 1.17 |
| Failure to follow train operation procedures | Demand of safety training | 0.58 | 0.18 | 1.15 |
| Failure to complete approval procedures | Demand of safety training | 0.58 | 0.18 | 1.15 |
| Failure to promptly and effectively rectify identified hidden danger | Demand of safety training | 0.58 | 0.18 | 1.15 |
| Insufficient safety oversight of subcontractors | Demand of safety training | 0.58 | 0.18 | 0.69 |
| Absence of a feasible construction plan | Inadequate on-site safety inspection | 0.29 | 0.18 | 2.30 |
| Failure to follow construction operation procedures | Inadequate identification of safety risks | 0.29 | 0.18 | 0.63 |
| Failure to follow dispatch operation procedures | Inadequate identification of safety risks | 0.29 | 0.18 | 2.30 |
| Lack of detailed train dispatching regulations | Inadequate identification of safety risks | 0.29 | 0.18 | 3.45 |
| Failure to set up safety warning signs | Inadequate on-site safety inspection | 0.29 | 0.18 | 1.73 |
| Failure to complete approval procedures | Inadequate identification of safety risks | 0.29 | 0.18 | 1.15 |
| Failure to carry out hidden danger identification | Inadequate on-site safety inspection | 0.29 | 0.18 | 2.30 |
| Absence of a safety risk management mechanism | Failure to follow construction operation procedures | 0.29 | 0.18 | 2.30 |
| Failure to carry out technical disclosure | Failure to follow construction operation procedures | 0.29 | 0.18 | 3.45 |
| Failure to promptly and effectively rectify identified hidden danger | Inadequate on-site safety inspection | 0.29 | 0.18 | 1.15 |
| Failure to promptly report accident information | Inadequate identification of safety risks | 0.29 | 0.18 | 1.73 |
| Failure to conduct regular emergency drills | Inadequate identification of safety risks | 0.29 | 0.18 | 1.73 |
| Inadequate identification of safety risks | Failure to follow construction operation procedures | 0.29 | 0.18 | 0.63 |
| Civil infrastructure failures | Inadequate identification of safety risks | 0.29 | 0.18 | 2.30 |
| Inadequate flood protection capability of safety equipment and facilities | Inadequate identification of safety risks | 0.29 | 0.18 | 3.45 |
| Drainage systems failures | Inadequate on-site safety inspection | 0.29 | 0.18 | 2.30 |
| Drainage systems failures | Failure to follow construction operation procedures | 0.29 | 0.18 | 2.30 |
| Maintenance personnel illegal operation | Inadequate on-site safety inspection | 0.29 | 0.18 | 1.73 |
| Poor quality control in construction project execution | Inadequate identification of safety risks | 0.29 | 0.18 | 2.30 |
| Dispatchers illegal operation | risk-taking mentality | 0.29 | 0.18 | 1.38 |
| Dispatchers illegal operation | Inadequate identification of safety risks | 0.29 | 0.18 | 1.38 |
| Lack of targeted content in safety training | Inadequate on-site safety inspection | 0.29 | 0.18 | 1.15 |
| Lack of targeted content in safety training | Inadequate identification of safety risks | 0.29 | 0.18 | 1.15 |
| Unclear safety responsibilities for employees | Inadequate enforcement of safety supervision and inspection systems | 0.45 | 0.18 | 1.34 |
| Failure to follow train operation procedures | Workplace audits and inspections | 0.45 | 0.18 | 1.12 |
| Failure to set up safety warning signs | Individual involvement in safety | 0.45 | 0.18 | 1.68 |
| Failure to complete approval procedures | Individual involvement in safety | 0.45 | 0.18 | 1.12 |
| Failure to promptly report accident information | Individual involvement in safety | 0.45 | 0.18 | 1.68 |
| Failure to conduct regular emergency drills | Individual involvement in safety | 0.45 | 0.18 | 1.68 |
| Drainage systems failures | Construction workers illegal operation | 0.45 | 0.18 | 2.24 |
| Power supply systems failures | Inadequate enforcement of safety supervision and inspection systems | 0.45 | 0.18 | 1.12 |
| Poor quality control in construction project execution | Workplace audits and inspections | 0.45 | 0.18 | 2.24 |
| Vehicle systems failures | Construction workers illegal operation | 0.45 | 0.18 | 2.24 |
| Unclear safety responsibilities for employees | Safety importance | 0.76 | 0.17 | 1.31 |
| Lack of targeted content in safety training | Safety importance | 0.76 | 0.17 | 1.09 |
| Construction workers illegal operation | Insufficient security knowledge | 0.32 | 0.17 | 0.37 |
| Designers illegal operation | Insufficient security knowledge | 0.32 | 0.17 | 3.17 |
| Unauthorized design modifications | Insufficient security knowledge | 0.32 | 0.17 | 3.17 |
| Station attendants illegal operation | Lack of targeted content in safety training | 0.16 | 0.17 | 6.33 |
| Unclear safety responsibilities for employees | Lack of targeted content in safety training | 0.16 | 0.17 | 1.27 |
| Inadequate coordination across emergency response plans | Lack of targeted content in safety training | 0.16 | 0.17 | 6.33 |
| Inadequate coordination across emergency response plans | Failure to promptly and effectively rectify identified hidden danger | 0.16 | 0.17 | 6.33 |
| Emergency response plan not updated in a timely manner | Lack of targeted content in safety training | 0.16 | 0.17 | 6.33 |
| Blind spots in hidden danger identification | Lack of targeted content in safety training | 0.16 | 0.17 | 0.49 |
| Incomplete signaling system | Lack of targeted content in safety training | 0.16 | 0.17 | 6.33 |
| Track and line systems failures | Failure to follow train operation procedures | 0.16 | 0.17 | 3.17 |
| Insufficient supervision of on-site operations | Lack of targeted content in safety training | 0.16 | 0.17 | 0.90 |
| Insufficient supervision of on-site operations | Failure to promptly and effectively rectify identified hidden danger | 0.16 | 0.17 | 0.90 |
| Missing documentation of on-site operational procedures | Failure to complete approval procedures | 0.16 | 0.17 | 3.17 |
| Absence of a hidden danger identification and mitigation system | Lack of targeted content in safety training | 0.16 | 0.17 | 3.17 |
| Absence of an on-site response plan | Failure to follow train operation procedures | 0.16 | 0.17 | 6.33 |
| Absence of a maintenance operation plan | Lack of targeted content in safety training | 0.16 | 0.17 | 6.33 |
| Absence of a feasible construction plan | Failure to promptly and effectively rectify identified hidden danger | 0.16 | 0.17 | 2.11 |
| Absence of operational procedures | Lack of targeted content in safety training | 0.16 | 0.17 | 3.17 |
| Failure to follow train operation procedures | Lack of targeted content in safety training | 0.16 | 0.17 | 1.06 |
| Failure to follow dispatch operation procedures | Lack of targeted content in safety training | 0.16 | 0.17 | 2.11 |
| Failure to follow dispatch operation procedures | Failure to promptly and effectively rectify identified hidden danger | 0.16 | 0.17 | 2.11 |
| Failure to enforce certification-based job qualification | Lack of targeted content in safety training | 0.16 | 0.17 | 6.33 |
| Failure to enforce certification-based job qualification | Failure to follow train operation procedures | 0.16 | 0.17 | 6.33 |
| Failure to enforce certification-based job qualification | Failure to promptly and effectively rectify identified hidden danger | 0.16 | 0.17 | 6.33 |
| Failure to effectively implement the all-staff safety responsibility system | Lack of targeted content in safety training | 0.16 | 0.17 | 0.49 |
| Lack of detailed train dispatching regulations | Lack of targeted content in safety training | 0.16 | 0.17 | 3.17 |
| Lack of detailed train dispatching regulations | Failure to promptly and effectively rectify identified hidden danger | 0.16 | 0.17 | 3.17 |
| Failure to set up safety warning signs | Failure to promptly and effectively rectify identified hidden danger | 0.16 | 0.17 | 1.58 |
| Failure to conduct risk assessment for equipment maintenance operations | Lack of targeted content in safety training | 0.16 | 0.17 | 6.33 |
| Absence of a safety risk management mechanism | Lack of targeted content in safety training | 0.16 | 0.17 | 2.11 |
| Failure to carry out technical disclosure | Failure to complete approval procedures | 0.16 | 0.17 | 3.17 |
| Failure to carry out technical disclosure | Failure to promptly and effectively rectify identified hidden danger | 0.16 | 0.17 | 3.17 |
| Failure to promptly and effectively rectify identified hidden danger | Lack of targeted content in safety training | 0.16 | 0.17 | 1.06 |
| Failure to promptly report accident information | Lack of targeted content in safety training | 0.16 | 0.17 | 1.58 |
| Failure to promptly report accident information | Failure to promptly and effectively rectify identified hidden danger | 0.16 | 0.17 | 1.58 |
| Failure to inform employees of existing safety risks and countermeasures | Lack of targeted content in safety training | 0.16 | 0.17 | 3.17 |
| Failure to conduct regular emergency drills | Failure to promptly and effectively rectify identified hidden danger | 0.16 | 0.17 | 1.58 |
| Drivers illegal operation | Lack of targeted content in safety training | 0.16 | 0.17 | 0.90 |
| Inadequate flood protection capability of safety equipment and facilities | Failure to promptly and effectively rectify identified hidden danger | 0.16 | 0.17 | 3.17 |
| Construction workers illegal operation | Lack of targeted content in safety training | 0.16 | 0.17 | 0.37 |
| Designers illegal operation | Lack of targeted content in safety training | 0.16 | 0.17 | 3.17 |
| Inadequate maintenance of equipment and facilities | Failure to promptly and effectively rectify identified hidden danger | 0.16 | 0.17 | 0.70 |
| risk-taking mentality | Lack of targeted content in safety training | 0.16 | 0.17 | 0.58 |
| Power supply systems failures | Failure to promptly and effectively rectify identified hidden danger | 0.16 | 0.17 | 1.06 |
| Poor quality control in construction project execution | Failure to promptly and effectively rectify identified hidden danger | 0.16 | 0.17 | 2.11 |
| Insufficient safety oversight of subcontractors | Lack of targeted content in safety training | 0.16 | 0.17 | 0.63 |
| Dispatchers illegal operation | Power supply systems failures | 0.16 | 0.17 | 1.27 |
| Dispatchers illegal operation | Failure to promptly and effectively rectify identified hidden danger | 0.16 | 0.17 | 1.27 |
| Insufficient security knowledge | Failure to promptly and effectively rectify identified hidden danger | 0.16 | 0.17 | 0.53 |
| Ineffective safety training outcomes | Failure to promptly and effectively rectify identified hidden danger | 0.16 | 0.17 | 0.40 |
| Lack of targeted content in safety training | Failure to promptly and effectively rectify identified hidden danger | 0.16 | 0.17 | 1.06 |
| Failure to set up safety warning signs | Safety of contractors and subsidiaries | 0.50 | 0.16 | 1.50 |
| Failure to promptly and effectively rectify identified hidden danger | Safety of contractors and subsidiaries | 0.50 | 0.16 | 1.00 |
| Inadequate maintenance of equipment and facilities | Safety of contractors and subsidiaries | 0.50 | 0.16 | 0.67 |
| Drainage systems failures | Safety of contractors and subsidiaries | 0.50 | 0.16 | 2.00 |
| Poor quality control in construction project execution | Safety of contractors and subsidiaries | 0.50 | 0.16 | 2.00 |
| Insufficient supervision of on-site operations | Blind spots in hidden danger identification | 0.34 | 0.15 | 0.84 |
| Failure to set up safety warning signs | Failure to effectively implement the all-staff safety responsibility system | 0.34 | 0.15 | 1.46 |
| Failure to promptly report accident information | Blind spots in hidden danger identification | 0.34 | 0.15 | 1.46 |
| Inadequate flood protection capability of safety equipment and facilities | Failure to effectively implement the all-staff safety responsibility system | 0.34 | 0.15 | 2.92 |
| Unauthorized design modifications | Blind spots in hidden danger identification | 0.34 | 0.15 | 2.92 |
| risk-taking mentality | Blind spots in hidden danger identification | 0.34 | 0.15 | 0.53 |
| Power supply systems failures | Blind spots in hidden danger identification | 0.34 | 0.15 | 0.97 |
| Power supply systems failures | Failure to effectively implement the all-staff safety responsibility system | 0.34 | 0.15 | 0.97 |
| Poor quality control in construction project execution | Blind spots in hidden danger identification | 0.34 | 0.15 | 1.95 |
| Poor quality control in construction project execution | Failure to effectively implement the all-staff safety responsibility system | 0.34 | 0.15 | 1.95 |
| Dispatchers illegal operation | Blind spots in hidden danger identification | 0.34 | 0.15 | 1.17 |
| Failure to complete approval procedures | Safety consciousness | 0.71 | 0.15 | 0.94 |
| Failure to conduct regular emergency drills | Safety consciousness | 0.71 | 0.15 | 1.41 |
| Inadequate maintenance of equipment and facilities | Safety consciousness | 0.71 | 0.15 | 0.63 |
| Drivers illegal operation | Luck-driven mentality | 0.37 | 0.14 | 0.78 |
| Inadequate flood protection capability of safety equipment and facilities | Facility satisfaction | 0.37 | 0.14 | 2.71 |
| Designers illegal operation | Luck-driven mentality | 0.37 | 0.14 | 2.71 |
| Unauthorized design modifications | Luck-driven mentality | 0.37 | 0.14 | 2.71 |
| Power supply systems failures | Luck-driven mentality | 0.37 | 0.14 | 0.90 |
| Lack of targeted content in safety training | Facility satisfaction | 0.37 | 0.14 | 0.90 |
| Track and line systems failures | Drivers illegal operation | 0.18 | 0.14 | 2.71 |
| Absence of a hidden danger identification and mitigation system | Insufficient supervision of on-site operations | 0.18 | 0.14 | 2.71 |
| Failure to set up safety warning signs | Insufficient supervision of on-site operations | 0.18 | 0.14 | 1.36 |
| Failure to carry out hidden danger identification | Insufficient supervision of on-site operations | 0.18 | 0.14 | 1.81 |
| Lack of safety training | Insufficient supervision of on-site operations | 0.18 | 0.14 | 1.81 |
| Failure to promptly and effectively rectify identified hidden danger | Insufficient supervision of on-site operations | 0.18 | 0.14 | 0.90 |
| Failure to inform employees of existing safety risks and countermeasures | Insufficient supervision of on-site operations | 0.18 | 0.14 | 2.71 |
| Inadequate identification of safety risks | Insufficient supervision of on-site operations | 0.18 | 0.14 | 0.49 |
| Drainage systems failures | Insufficient supervision of on-site operations | 0.18 | 0.14 | 1.81 |
| Maintenance personnel illegal operation | Insufficient supervision of on-site operations | 0.18 | 0.14 | 1.36 |
| Insufficient safety oversight of subcontractors | Insufficient supervision of on-site operations | 0.18 | 0.14 | 0.54 |
| Insufficient security knowledge | Insufficient supervision of on-site operations | 0.18 | 0.14 | 0.45 |
| Failure to set up safety warning signs | Safety importance | 0.76 | 0.14 | 1.31 |
| Failure to promptly report accident information | Safety importance | 0.76 | 0.14 | 1.31 |
| Failure to conduct regular emergency drills | Safety importance | 0.76 | 0.14 | 1.31 |
| Unclear safety responsibilities for employees | Demand of safety training | 0.58 | 0.14 | 1.04 |
| Lack of safety training | Demand of safety training | 0.58 | 0.14 | 1.73 |
| Absence of a safety risk management mechanism | Demand of safety training | 0.58 | 0.14 | 1.73 |
| Failure to conduct regular emergency drills | Demand of safety training | 0.58 | 0.14 | 1.30 |
| Absence of a feasible construction plan | Inadequate enforcement of safety supervision and inspection systems | 0.45 | 0.12 | 1.49 |
| Failure to follow dispatch operation procedures | Inadequate enforcement of safety supervision and inspection systems | 0.45 | 0.12 | 1.49 |
| Failure to follow dispatch operation procedures | Individual involvement in safety | 0.45 | 0.12 | 1.49 |
| Failure to set up safety warning signs | Inadequate enforcement of safety supervision and inspection systems | 0.45 | 0.12 | 1.12 |
| Failure to carry out hidden danger identification | Workplace audits and inspections | 0.45 | 0.12 | 1.49 |
| Failure to carry out hidden danger identification | Individual involvement in safety | 0.45 | 0.12 | 1.49 |
| Lack of safety training | Inadequate enforcement of safety supervision and inspection systems | 0.45 | 0.12 | 1.49 |
| Absence of a safety risk management mechanism | Inadequate enforcement of safety supervision and inspection systems | 0.45 | 0.12 | 1.49 |
| Absence of a safety risk management mechanism | Workplace audits and inspections | 0.45 | 0.12 | 1.49 |
| Failure to promptly report accident information | Workplace audits and inspections | 0.45 | 0.12 | 1.12 |
| Failure to inform employees of existing safety risks and countermeasures | Inadequate enforcement of safety supervision and inspection systems | 0.45 | 0.12 | 2.24 |
| Failure to conduct regular emergency drills | Inadequate enforcement of safety supervision and inspection systems | 0.45 | 0.12 | 1.12 |
| Inadequate flood protection capability of safety equipment and facilities | Inadequate enforcement of safety supervision and inspection systems | 0.45 | 0.12 | 2.24 |
| Inadequate flood protection capability of safety equipment and facilities | Workplace audits and inspections | 0.45 | 0.12 | 2.24 |
| Maintenance personnel illegal operation | Inadequate enforcement of safety supervision and inspection systems | 0.45 | 0.12 | 1.12 |
| Poor quality control in construction project execution | Inadequate enforcement of safety supervision and inspection systems | 0.45 | 0.12 | 1.49 |
| Dispatchers illegal operation | Inadequate enforcement of safety supervision and inspection systems | 0.45 | 0.12 | 0.89 |
| Lack of targeted content in safety training | Inadequate enforcement of safety supervision and inspection systems | 0.45 | 0.12 | 0.75 |
| Lack of targeted content in safety training | Workplace audits and inspections | 0.45 | 0.12 | 0.75 |
| Lack of targeted content in safety training | Individual involvement in safety | 0.45 | 0.12 | 0.75 |
| Dispatchers illegal operation | Insufficient safety consciousness | 0.68 | 0.12 | 0.88 |
| Vehicle systems failures | Insufficient safety consciousness | 0.68 | 0.12 | 1.46 |
| Failure to follow dispatch operation procedures | Safety consciousness | 0.71 | 0.11 | 1.41 |
| Absence of a safety risk management mechanism | Safety consciousness | 0.71 | 0.11 | 1.41 |
| Failure to promptly report accident information | Safety consciousness | 0.71 | 0.11 | 1.06 |
| Drainage systems failures | Inadequate maintenance of equipment and facilities | 0.24 | 0.11 | 1.41 |
| Vehicle systems failures | Inadequate maintenance of equipment and facilities | 0.24 | 0.11 | 1.41 |
| Absence of a feasible construction plan | Safety of contractors and subsidiaries | 0.50 | 0.11 | 1.33 |
| Inadequate flood protection capability of safety equipment and facilities | Safety of contractors and subsidiaries | 0.50 | 0.11 | 2.00 |
| Absence of a feasible construction plan | Safety importance | 0.76 | 0.10 | 1.31 |
| Failure to follow dispatch operation procedures | Safety importance | 0.76 | 0.10 | 1.31 |
| Failure to carry out hidden danger identification | Safety importance | 0.76 | 0.10 | 1.31 |
| Lack of safety training | Safety importance | 0.76 | 0.10 | 1.31 |
| Absence of a safety risk management mechanism | Safety importance | 0.76 | 0.10 | 1.31 |
| Inadequate maintenance of equipment and facilities | Safety importance | 0.76 | 0.10 | 0.44 |
| Insufficient safety consciousness | Insufficient supervision of on-site operations | 0.18 | 1.00 | 1.46 |
| Drivers illegal operation | Failure to follow train operation procedures | 0.16 | 1.00 | 5.43 |
| Insufficient safety consciousness | Failure to promptly and effectively rectify identified hidden danger | 0.16 | 1.00 | 1.46 |
| Insufficient safety consciousness | Insufficient safety oversight of subcontractors | 0.26 | 0.90 | 1.32 |
| Construction workers illegal operation | Failure to set up safety warning signs | 0.11 | 1.00 | 2.24 |
| Insufficient safety consciousness | Failure to set up safety warning signs | 0.11 | 1.00 | 1.46 |
| Construction workers illegal operation | Insufficient safety oversight of subcontractors | 0.26 | 0.80 | 1.79 |
| Insufficient safety consciousness | Unclear safety responsibilities for employees | 0.13 | 0.80 | 1.17 |
| Insufficient safety consciousness | Ineffective safety training outcomes | 0.42 | 0.75 | 1.10 |
| Failure to promptly report accident information | Safety responsibility of managers | 0.11 | 0.75 | 7.13 |
| Failure to conduct regular emergency drills | Safety responsibility of managers | 0.11 | 0.75 | 7.13 |
| Failure to conduct regular emergency drills | Beliefs of zero-accident | 0.11 | 0.75 | 7.13 |
| Dispatchers illegal operation | Failure to conduct regular emergency drills | 0.11 | 0.75 | 5.70 |
| Insufficient security knowledge | Failure to conduct regular emergency drills | 0.11 | 0.75 | 2.38 |
| Inadequate enforcement of safety supervision and inspection systems | Ineffective safety training outcomes | 0.42 | 0.69 | 1.54 |
| Inadequate enforcement of safety supervision and inspection systems | Enforcement of safety rules | 0.66 | 0.60 | 1.34 |
| Luck-driven mentality | Insufficient safety oversight of subcontractors | 0.26 | 0.60 | 1.63 |
| Ineffective safety training outcomes | Insufficient safety oversight of subcontractors | 0.26 | 0.60 | 1.43 |
| Inadequate enforcement of safety supervision and inspection systems | Insufficient safety oversight of subcontractors | 0.26 | 0.60 | 1.34 |
| Blind spots in hidden danger identification | Unclear safety responsibilities for employees | 0.13 | 0.60 | 1.75 |
| Failure to promptly report accident information | Emergency capability | 0.13 | 0.60 | 5.70 |
| Failure to conduct regular emergency drills | Emergency capability | 0.13 | 0.60 | 5.70 |
| Failure to conduct regular emergency drills | Responsibilities for safety in every department | 0.13 | 0.60 | 5.70 |
| Inadequate identification of safety risks | Responsibilities for safety in every department | 0.13 | 0.60 | 2.07 |
| Drivers illegal operation | Dispatchers illegal operation | 0.13 | 0.60 | 3.26 |
| Luck-driven mentality | Unclear safety responsibilities for employees | 0.13 | 0.60 | 1.63 |
| Insufficient security knowledge | Unclear safety responsibilities for employees | 0.13 | 0.60 | 1.90 |
| Inadequate enforcement of safety supervision and inspection systems | Unclear safety responsibilities for employees | 0.13 | 0.60 | 1.34 |
| Construction workers illegal operation | Ineffective safety training outcomes | 0.42 | 0.56 | 1.26 |
| Ineffective safety training outcomes | Demand of safety training | 0.58 | 0.50 | 1.19 |
| Blind spots in hidden danger identification | Facility satisfaction | 0.37 | 0.50 | 1.46 |
| Construction workers illegal operation | Luck-driven mentality | 0.37 | 0.50 | 1.12 |
| Ineffective safety training outcomes | Facility satisfaction | 0.37 | 0.50 | 1.19 |
| Luck-driven mentality | Insufficient security knowledge | 0.32 | 0.50 | 1.36 |
| Insufficient safety consciousness | Insufficient security knowledge | 0.32 | 0.50 | 0.73 |
| Failure to complete approval procedures | Failure to promptly and effectively rectify identified hidden danger | 0.16 | 0.50 | 3.17 |
| Inadequate identification of safety risks | Failure to promptly and effectively rectify identified hidden danger | 0.16 | 0.50 | 1.73 |
| Dispatchers illegal operation | Failure to follow train operation procedures | 0.16 | 0.50 | 3.80 |
| Insufficient safety consciousness | Lack of targeted content in safety training | 0.16 | 0.50 | 0.73 |
| Unclear safety responsibilities for employees | Safety responsibility of managers | 0.11 | 0.50 | 3.80 |
| Blind spots in hidden danger identification | Safety responsibility of managers | 0.11 | 0.50 | 1.46 |
| Blind spots in hidden danger identification | Beliefs of zero-accident | 0.11 | 0.50 | 1.46 |
| Failure to follow train operation procedures | Failure to conduct regular emergency drills | 0.11 | 0.50 | 3.17 |
| Failure to follow train operation procedures | Safety responsibility of managers | 0.11 | 0.50 | 3.17 |
| Failure to follow dispatch operation procedures | Failure to conduct regular emergency drills | 0.11 | 0.50 | 6.33 |
| Failure to follow dispatch operation procedures | Safety responsibility of managers | 0.11 | 0.50 | 6.33 |
| Lack of detailed train dispatching regulations | Safety responsibility of managers | 0.11 | 0.50 | 9.50 |
| Failure to evacuate passengers in a timely manner | Failure to promptly report accident information | 0.11 | 0.50 | 9.50 |
| Failure to promptly report accident information | Failure to conduct regular emergency drills | 0.11 | 0.50 | 4.75 |
| Inadequate identification of safety risks | Safety responsibility of managers | 0.11 | 0.50 | 1.73 |
| Drivers illegal operation | Failure to conduct regular emergency drills | 0.11 | 0.50 | 2.71 |
| Insufficient safety consciousness | Failure to promptly report accident information | 0.11 | 0.50 | 0.73 |
| Insufficient safety consciousness | Failure to conduct regular emergency drills | 0.11 | 0.50 | 0.73 |
| Lack of targeted content in safety training | Failure to conduct regular emergency drills | 0.11 | 0.50 | 3.17 |
| Lack of targeted content in safety training | Safety responsibility of managers | 0.11 | 0.50 | 3.17 |
| Failure to effectively implement the all-staff safety responsibility system | Enforcement of safety rules | 0.66 | 0.48 | 1.40 |
| Ineffective safety training outcomes | Enforcement of safety rules | 0.66 | 0.44 | 1.05 |
| Blind spots in hidden danger identification | Ineffective safety training outcomes | 0.42 | 0.44 | 1.28 |
| Failure to effectively implement the all-staff safety responsibility system | Ineffective safety training outcomes | 0.42 | 0.44 | 1.28 |
| Inadequate maintenance of equipment and facilities | Ineffective safety training outcomes | 0.42 | 0.44 | 1.85 |
| Failure to follow construction operation procedures | Enforcement of safety rules | 0.66 | 0.40 | 1.38 |
| Inadequate on-site safety inspection | Insufficient safety oversight of subcontractors | 0.26 | 0.40 | 1.38 |
| Failure to follow construction operation procedures | Insufficient safety oversight of subcontractors | 0.26 | 0.40 | 1.38 |
| Insufficient security knowledge | Insufficient safety oversight of subcontractors | 0.26 | 0.40 | 1.27 |
| Unclear safety responsibilities for employees | Responsibilities for safety in every department | 0.13 | 0.40 | 3.04 |
| Insufficient supervision of on-site operations | Unclear safety responsibilities for employees | 0.13 | 0.40 | 2.17 |
| Failure to follow construction operation procedures | Unclear safety responsibilities for employees | 0.13 | 0.40 | 1.38 |
| Lack of detailed train dispatching regulations | Responsibilities for safety in every department | 0.13 | 0.40 | 7.60 |
| Failure to evacuate passengers in a timely manner | Emergency capability | 0.13 | 0.40 | 7.60 |
| Failure to promptly report accident information | Unclear safety responsibilities for employees | 0.13 | 0.40 | 3.80 |
| Failure to inform employees of existing safety risks and countermeasures | Unclear safety responsibilities for employees | 0.13 | 0.40 | 7.60 |
| Failure to conduct regular emergency drills | Unclear safety responsibilities for employees | 0.13 | 0.40 | 3.80 |
| Maintenance personnel illegal operation | Unclear safety responsibilities for employees | 0.13 | 0.40 | 3.80 |
| Poor quality control in construction project execution | Responsibilities for safety in every department | 0.13 | 0.40 | 5.07 |
| Insufficient safety oversight of subcontractors | Unclear safety responsibilities for employees | 0.13 | 0.40 | 1.52 |
| Insufficient safety oversight of subcontractors | Responsibilities for safety in every department | 0.13 | 0.40 | 1.52 |
| Dispatchers illegal operation | Unclear safety responsibilities for employees | 0.13 | 0.40 | 3.04 |
| Lack of targeted content in safety training | Responsibilities for safety in every department | 0.13 | 0.40 | 2.53 |
| Inadequate enforcement of safety supervision and inspection systems | Responsibilities for safety in every department | 0.13 | 0.40 | 0.89 |
| Inadequate on-site safety inspection | Ineffective safety training outcomes | 0.42 | 0.38 | 1.30 |
| Luck-driven mentality | Ineffective safety training outcomes | 0.42 | 0.38 | 1.02 |
| Insufficient safety oversight of subcontractors | Ineffective safety training outcomes | 0.42 | 0.38 | 1.43 |
| Inadequate on-site safety inspection | Enforcement of safety rules | 0.66 | 0.36 | 1.24 |
| Blind spots in hidden danger identification | Enforcement of safety rules | 0.66 | 0.32 | 0.94 |
| Failure to follow construction operation procedures | Ineffective safety training outcomes | 0.42 | 0.31 | 1.08 |
| Inadequate identification of safety risks | Ineffective safety training outcomes | 0.42 | 0.31 | 1.08 |
| Blind spots in hidden danger identification | Insufficient safety oversight of subcontractors | 0.26 | 0.30 | 0.88 |
| Inadequate identification of safety risks | Insufficient safety oversight of subcontractors | 0.26 | 0.30 | 1.04 |
| Inadequate maintenance of equipment and facilities | Insufficient safety oversight of subcontractors | 0.26 | 0.30 | 1.27 |
| Poor quality control in construction project execution | Insufficient safety oversight of subcontractors | 0.26 | 0.30 | 3.80 |
| Insufficient supervision of on-site operations | Ineffective safety training outcomes | 0.42 | 0.25 | 1.36 |
| Signaling systems failures | Insufficient security knowledge | 0.32 | 0.25 | 1.90 |
| Drivers illegal operation | Insufficient security knowledge | 0.32 | 0.25 | 1.36 |
| Maintenance personnel illegal operation | Insufficient security knowledge | 0.32 | 0.25 | 2.38 |
| Station attendants illegal operation | Failure to conduct regular emergency drills | 0.11 | 0.25 | 9.50 |
| Inadequate coordination across emergency response plans | Failure to conduct regular emergency drills | 0.11 | 0.25 | 9.50 |
| Inadequate coordination across emergency response plans | Safety responsibility of managers | 0.11 | 0.25 | 9.50 |
| Emergency response plan not approved through formal review | Safety responsibility of managers | 0.11 | 0.25 | 9.50 |
| Emergency response plan not updated in a timely manner | Failure to conduct regular emergency drills | 0.11 | 0.25 | 9.50 |
| Emergency response plan not updated in a timely manner | Safety responsibility of managers | 0.11 | 0.25 | 9.50 |
| Insufficient content in the emergency response plan | Failure to conduct regular emergency drills | 0.11 | 0.25 | 9.50 |
| Insufficient content in the emergency response plan | Safety responsibility of managers | 0.11 | 0.25 | 9.50 |
| Inadequate content in the on-site response plan | Safety responsibility of managers | 0.11 | 0.25 | 9.50 |
| Absence of a hidden danger identification and mitigation system | Safety responsibility of managers | 0.11 | 0.25 | 4.75 |
| Absence of a hidden danger identification and mitigation system | Beliefs of zero-accident | 0.11 | 0.25 | 4.75 |
| Absence of an on-site response plan | Safety responsibility of managers | 0.11 | 0.25 | 9.50 |
| Absence of a maintenance operation plan | Safety responsibility of managers | 0.11 | 0.25 | 9.50 |
| Absence of operational procedures | Safety responsibility of managers | 0.11 | 0.25 | 4.75 |
| Absence of operational procedures | Beliefs of zero-accident | 0.11 | 0.25 | 4.75 |
| Failure to enforce certification-based job qualification | Safety responsibility of managers | 0.11 | 0.25 | 9.50 |
| Failure to effectively implement the all-staff safety responsibility system | Safety responsibility of managers | 0.11 | 0.25 | 0.73 |
| Failure to activate emergency response | Failure to promptly report accident information | 0.11 | 0.25 | 9.50 |
| Failure to activate emergency response | Safety responsibility of managers | 0.11 | 0.25 | 9.50 |
| Failure to carry out hidden danger identification | Safety responsibility of managers | 0.11 | 0.25 | 3.17 |
| Failure to carry out hidden danger identification | Beliefs of zero-accident | 0.11 | 0.25 | 3.17 |
| Failure to conduct risk assessment for equipment maintenance operations | Safety responsibility of managers | 0.11 | 0.25 | 9.50 |
| Absence of a safety risk management mechanism | Safety responsibility of managers | 0.11 | 0.25 | 3.17 |
| Failure to promptly and effectively rectify identified hidden danger | Safety responsibility of managers | 0.11 | 0.25 | 1.58 |
| Failure to evacuate passengers in a timely manner | Safety responsibility of managers | 0.11 | 0.25 | 4.75 |
| Failure to issue timely suspension instructions for the rail network | Failure to promptly report accident information | 0.11 | 0.25 | 9.50 |
| Failure to issue timely suspension instructions for the rail network | Safety responsibility of managers | 0.11 | 0.25 | 9.50 |
| Absence of emergency response plans | Safety responsibility of managers | 0.11 | 0.25 | 9.50 |
| The absence of unified on-site command and dispatch by leadership | Failure to promptly report accident information | 0.11 | 0.25 | 9.50 |
| The absence of unified on-site command and dispatch by leadership | Safety responsibility of managers | 0.11 | 0.25 | 9.50 |
| Power supply systems failures | Maintenance personnel illegal operation | 0.11 | 0.25 | 1.58 |
| Poor quality control in construction project execution | Safety responsibility of managers | 0.11 | 0.25 | 3.17 |
| Insufficient safety oversight of subcontractors | Safety responsibility of managers | 0.11 | 0.25 | 0.95 |
| Insufficient coordination capacity in dispatch command | Safety responsibility of managers | 0.11 | 0.25 | 9.50 |
| Inadequate enforcement of safety supervision and inspection systems | Safety responsibility of managers | 0.11 | 0.25 | 0.56 |
| Inadequate enforcement of safety supervision and inspection systems | Beliefs of zero-accident | 0.11 | 0.25 | 0.56 |
| Failure to complete approval procedures | Enforcement of safety rules | 0.66 | 0.24 | 1.52 |
| Failure to promptly and effectively rectify identified hidden danger | Enforcement of safety rules | 0.66 | 0.24 | 1.52 |
| Insufficient safety oversight of subcontractors | Enforcement of safety rules | 0.66 | 0.24 | 0.91 |
| Failure to follow train operation procedures | Enforcement of safety rules | 0.66 | 0.20 | 1.27 |
| Unclear safety responsibilities for employees | Insufficient safety oversight of subcontractors | 0.26 | 0.20 | 1.52 |
| Failure to set up safety warning signs | Insufficient safety oversight of subcontractors | 0.26 | 0.20 | 1.90 |
| Failure to complete approval procedures | Insufficient safety oversight of subcontractors | 0.26 | 0.20 | 1.27 |
| Failure to promptly and effectively rectify identified hidden danger | Insufficient safety oversight of subcontractors | 0.26 | 0.20 | 1.27 |
| Inadequate flood protection capability of safety equipment and facilities | Insufficient safety oversight of subcontractors | 0.26 | 0.20 | 3.80 |
| Unauthorized design modifications | Insufficient safety oversight of subcontractors | 0.26 | 0.20 | 3.80 |
| Drainage systems failures | Insufficient safety oversight of subcontractors | 0.26 | 0.20 | 2.53 |
| risk-taking mentality | Insufficient safety oversight of subcontractors | 0.26 | 0.20 | 0.69 |
| Vehicle systems failures | Insufficient safety oversight of subcontractors | 0.26 | 0.20 | 2.53 |
| Inadequate coordination across emergency response plans | Emergency capability | 0.13 | 0.20 | 7.60 |
| Emergency response plan not approved through formal review | Unclear safety responsibilities for employees | 0.13 | 0.20 | 7.60 |
| Emergency response plan not updated in a timely manner | Emergency capability | 0.13 | 0.20 | 7.60 |
| Insufficient content in the emergency response plan | Unclear safety responsibilities for employees | 0.13 | 0.20 | 7.60 |
| Inadequate content in the on-site response plan | Unclear safety responsibilities for employees | 0.13 | 0.20 | 7.60 |
| Inadequate on-site safety inspection | Unclear safety responsibilities for employees | 0.13 | 0.20 | 0.69 |
| Inadequate on-site safety inspection | Responsibilities for safety in every department | 0.13 | 0.20 | 0.69 |
| Absence of a hidden danger identification and mitigation system | Unclear safety responsibilities for employees | 0.13 | 0.20 | 3.80 |
| Absence of a hidden danger identification and mitigation system | Responsibilities for safety in every department | 0.13 | 0.20 | 3.80 |
| Absence of an on-site response plan | Unclear safety responsibilities for employees | 0.13 | 0.20 | 7.60 |
| Absence of an on-site response plan | Responsibilities for safety in every department | 0.13 | 0.20 | 7.60 |
| Absence of a maintenance operation plan | Responsibilities for safety in every department | 0.13 | 0.20 | 7.60 |
| Absence of operational procedures | Unclear safety responsibilities for employees | 0.13 | 0.20 | 3.80 |
| Absence of operational procedures | Responsibilities for safety in every department | 0.13 | 0.20 | 3.80 |
| Failure to follow train operation procedures | Unclear safety responsibilities for employees | 0.13 | 0.20 | 1.27 |
| Failure to follow dispatch operation procedures | Unclear safety responsibilities for employees | 0.13 | 0.20 | 2.53 |
| Lack of detailed train dispatching regulations | Unclear safety responsibilities for employees | 0.13 | 0.20 | 3.80 |
| Failure to activate emergency response | Unclear safety responsibilities for employees | 0.13 | 0.20 | 7.60 |
| Failure to activate emergency response | Emergency capability | 0.13 | 0.20 | 7.60 |
| Failure to carry out hidden danger identification | Unclear safety responsibilities for employees | 0.13 | 0.20 | 2.53 |
| Failure to carry out hidden danger identification | Responsibilities for safety in every department | 0.13 | 0.20 | 2.53 |
| Failure to conduct risk assessment for equipment maintenance operations | Responsibilities for safety in every department | 0.13 | 0.20 | 7.60 |
| Lack of safety training | Unclear safety responsibilities for employees | 0.13 | 0.20 | 2.53 |
| Absence of a safety risk management mechanism | Unclear safety responsibilities for employees | 0.13 | 0.20 | 2.53 |
| Absence of a safety risk management mechanism | Responsibilities for safety in every department | 0.13 | 0.20 | 2.53 |
| Failure to carry out technical disclosure | Unclear safety responsibilities for employees | 0.13 | 0.20 | 3.80 |
| Failure to evacuate passengers in a timely manner | Unclear safety responsibilities for employees | 0.13 | 0.20 | 3.80 |
| Failure to issue timely suspension instructions for the rail network | Unclear safety responsibilities for employees | 0.13 | 0.20 | 7.60 |
| Failure to issue timely suspension instructions for the rail network | Emergency capability | 0.13 | 0.20 | 7.60 |
| Inadequate identification of safety risks | Unclear safety responsibilities for employees | 0.13 | 0.20 | 0.69 |
| Absence of emergency response plans | Unclear safety responsibilities for employees | 0.13 | 0.20 | 7.60 |
| Absence of emergency response plans | Emergency capability | 0.13 | 0.20 | 7.60 |
| Absence of emergency response plans | Responsibilities for safety in every department | 0.13 | 0.20 | 7.60 |
| Drivers illegal operation | Unclear safety responsibilities for employees | 0.13 | 0.20 | 1.09 |
| Inadequate flood protection capability of safety equipment and facilities | Responsibilities for safety in every department | 0.13 | 0.20 | 3.80 |
| Construction workers illegal operation | Unclear safety responsibilities for employees | 0.13 | 0.20 | 0.45 |
| Inadequate maintenance of equipment and facilities | Responsibilities for safety in every department | 0.13 | 0.20 | 0.84 |
| Unauthorized design modifications | Unclear safety responsibilities for employees | 0.13 | 0.20 | 3.80 |
| Drainage systems failures | Responsibilities for safety in every department | 0.13 | 0.20 | 2.53 |
| The absence of unified on-site command and dispatch by leadership | Emergency capability | 0.13 | 0.20 | 7.60 |
| Failure to implement shift handover procedures | Unclear safety responsibilities for employees | 0.13 | 0.20 | 7.60 |
| Incomplete power supply system | Unclear safety responsibilities for employees | 0.13 | 0.20 | 7.60 |
| Poor quality control in construction project execution | Unclear safety responsibilities for employees | 0.13 | 0.20 | 2.53 |
| Insufficient coordination capacity in dispatch command | Unclear safety responsibilities for employees | 0.13 | 0.20 | 7.60 |
| Insufficient coordination capacity in dispatch command | Emergency capability | 0.13 | 0.20 | 7.60 |
| Insufficient coordination capacity in dispatch command | Responsibilities for safety in every department | 0.13 | 0.20 | 7.60 |
| Dispatchers illegal operation | Signaling systems failures | 0.13 | 0.20 | 1.52 |
| Ineffective safety training outcomes | Unclear safety responsibilities for employees | 0.13 | 0.20 | 0.48 |
| Ineffective safety training outcomes | Responsibilities for safety in every department | 0.13 | 0.20 | 0.48 |
| Lack of targeted content in safety training | Unclear safety responsibilities for employees | 0.13 | 0.20 | 1.27 |
| Civil infrastructure failures | Ineffective safety training outcomes | 0.42 | 0.19 | 2.38 |
| Drivers illegal operation | Ineffective safety training outcomes | 0.42 | 0.19 | 1.02 |
| risk-taking mentality | Ineffective safety training outcomes | 0.42 | 0.19 | 0.65 |
| Insufficient security knowledge | Ineffective safety training outcomes | 0.42 | 0.19 | 0.59 |
| Failure to set up safety warning signs | Enforcement of safety rules | 0.66 | 0.16 | 1.52 |
| Lack of targeted content in safety training | Enforcement of safety rules | 0.66 | 0.16 | 1.01 |
| Signaling systems failures | Ineffective safety training outcomes | 0.42 | 0.13 | 0.95 |
| Absence of a feasible construction plan | Ineffective safety training outcomes | 0.42 | 0.13 | 1.58 |
| Failure to follow train operation procedures | Ineffective safety training outcomes | 0.42 | 0.13 | 0.79 |
| Failure to complete approval procedures | Ineffective safety training outcomes | 0.42 | 0.13 | 0.79 |
| Absence of a safety risk management mechanism | Ineffective safety training outcomes | 0.42 | 0.13 | 1.58 |
| Inadequate flood protection capability of safety equipment and facilities | Ineffective safety training outcomes | 0.42 | 0.13 | 2.38 |
| Drainage systems failures | Ineffective safety training outcomes | 0.42 | 0.13 | 1.58 |
| Power supply systems failures | Ineffective safety training outcomes | 0.42 | 0.13 | 0.79 |
| Poor quality control in construction project execution | Ineffective safety training outcomes | 0.42 | 0.13 | 1.58 |
| Failure to carry out hidden danger identification | Enforcement of safety rules | 0.66 | 0.12 | 1.52 |
| Absence of a safety risk management mechanism | Enforcement of safety rules | 0.66 | 0.12 | 1.52 |
| Inadequate maintenance of equipment and facilities | Enforcement of safety rules | 0.66 | 0.12 | 0.51 |
| Signaling systems failures | Insufficient safety oversight of subcontractors | 0.26 | 0.10 | 0.76 |
| Incomplete signaling system | Insufficient safety oversight of subcontractors | 0.26 | 0.10 | 3.80 |
| Insufficient supervision of on-site operations | Insufficient safety oversight of subcontractors | 0.26 | 0.10 | 0.54 |
| Absence of a hidden danger identification and mitigation system | Insufficient safety oversight of subcontractors | 0.26 | 0.10 | 1.90 |
| Absence of a feasible construction plan | Insufficient safety oversight of subcontractors | 0.26 | 0.10 | 1.27 |
| Failure to activate emergency response | Insufficient safety oversight of subcontractors | 0.26 | 0.10 | 3.80 |
| Failure to carry out hidden danger identification | Insufficient safety oversight of subcontractors | 0.26 | 0.10 | 1.27 |
| Absence of a safety risk management mechanism | Insufficient safety oversight of subcontractors | 0.26 | 0.10 | 1.27 |
| Failure to carry out technical disclosure | Insufficient safety oversight of subcontractors | 0.26 | 0.10 | 1.90 |
| Failure to promptly report accident information | Insufficient safety oversight of subcontractors | 0.26 | 0.10 | 0.95 |
| Absence of emergency response plans | Insufficient safety oversight of subcontractors | 0.26 | 0.10 | 3.80 |
| Civil infrastructure failures | Insufficient safety oversight of subcontractors | 0.26 | 0.10 | 1.27 |
| Designers illegal operation | Insufficient safety oversight of subcontractors | 0.26 | 0.10 | 1.90 |
| Power supply systems failures | Insufficient safety oversight of subcontractors | 0.26 | 0.10 | 0.63 |
| Lack of targeted content in safety training | Insufficient safety oversight of subcontractors | 0.26 | 0.10 | 0.63 |
