# Supplementary material for: Comprehensive analysis method of key risk factors in the subway operation accident by complex network and accident data
Source: PLoS One. 2026 Sep 18;21(9):e0358549. doi: 10.1371/journal.pone.0358549 (PMC13588349; doi:10.1371/journal.pone.0358549)
Supplement: Supplementary file 2 [file pone.0358549.s002.docx]

**Supplementary file 2**

**Results of node degree and node strength of risk factors**

| **Factors** | **In-degree** | **Out-degree** | **Total degree** | **In-strength** | **Out-strength** | **Total strength** |
| --- | --- | --- | --- | --- | --- | --- |
| A6 | 0 | 33 | 33 | 0 | 203.978404 | 203.978404 |
| B1 | 10 | 41 | 51 | 18.711099 | 152.295663 | 171.006762 |
| B12 | 16 | 36 | 52 | 22.505276 | 93.589586 | 116.094862 |
| B29 | 13 | 12 | 25 | 42.734597 | 66.936722 | 109.671319 |
| A9 | 0 | 22 | 22 | 0 | 87.437198 | 87.437198 |
| B19 | 15 | 31 | 46 | 21.12284 | 64.947206 | 86.070046 |
| B40 | 19 | 36 | 55 | 21.758563 | 63.505724 | 85.264287 |
| B36 | 14 | 5 | 19 | 37.007163 | 38.730769 | 75.737932 |
| A14 | 0 | 10 | 10 | 0 | 72.2 | 72.2 |
| B15 | 21 | 26 | 47 | 25.612152 | 41.94708 | 67.559232 |
| B5 | 20 | 32 | 52 | 23.207845 | 42.450567 | 65.658412 |
| B42 | 16 | 22 | 38 | 21.644952 | 41.01786 | 62.662811 |
| B2 | 18 | 23 | 41 | 23.159819 | 36.194139 | 59.353958 |
| B10 | 16 | 6 | 22 | 26.018414 | 28.228571 | 54.246985 |
| B41 | 17 | 21 | 38 | 19.515965 | 32.479435 | 51.995399 |
| B18 | 18 | 22 | 40 | 21.169088 | 29.276392 | 50.44548 |
| B7 | 18 | 11 | 29 | 24.324031 | 24.940738 | 49.264768 |
| B14 | 15 | 25 | 40 | 15.072142 | 32.217399 | 47.289541 |
| B31 | 5 | 0 | 5 | 39.266667 | 0 | 39.266667 |
| B39 | 5 | 0 | 5 | 38 | 0 | 38 |
| C1 | 15 | 10 | 25 | 17.933423 | 18.744119 | 36.677542 |
| D1 | 15 | 1 | 16 | 32.882955 | 3.257143 | 36.140098 |
| B33 | 5 | 0 | 5 | 36.1 | 0 | 36.1 |
| B48 | 14 | 0 | 14 | 35.716168 | 0 | 35.716168 |
| B26 | 15 | 7 | 22 | 17.1298 | 18.577778 | 35.707578 |
| B38 | 4 | 0 | 4 | 34.2 | 0 | 34.2 |
| D2 | 15 | 2 | 17 | 27.577518 | 5.971429 | 33.548947 |
| B32 | 4 | 0 | 4 | 32.933333 | 0 | 32.933333 |
| B23 | 11 | 0 | 11 | 32.926385 | 0 | 32.926385 |
| B44 | 16 | 2 | 18 | 26.155259 | 6.333333 | 32.488592 |
| B8 | 4 | 0 | 4 | 32.3 | 0 | 32.3 |
| C3 | 13 | 10 | 23 | 13.927061 | 18.269348 | 32.196409 |
| B35 | 4 | 0 | 4 | 31.033333 | 0 | 31.033333 |
| B6 | 6 | 0 | 6 | 30.687879 | 0 | 30.687879 |
| B13 | 4 | 0 | 4 | 28.5 | 0 | 28.5 |
| B17 | 14 | 0 | 14 | 28.061939 | 0 | 28.061939 |
| A11 | 0 | 19 | 19 | 0 | 27.756888 | 27.756888 |
| B9 | 10 | 0 | 10 | 27.423397 | 0 | 27.423397 |
| B30 | 3 | 0 | 3 | 26.6 | 0 | 26.6 |
| D8 | 3 | 0 | 3 | 26.6 | 0 | 26.6 |
| A1 | 0 | 23 | 23 | 0 | 26.554591 | 26.554591 |
| D4 | 17 | 2 | 19 | 21.629438 | 4.470588 | 26.100026 |
| A7 | 0 | 19 | 19 | 0 | 25.921381 | 25.921381 |
| B37 | 4 | 0 | 4 | 25.65 | 0 | 25.65 |
| B21 | 7 | 0 | 7 | 24.880952 | 0 | 24.880952 |
| C2 | 15 | 7 | 22 | 16.440023 | 7.984198 | 24.424221 |
| B25 | 3 | 0 | 3 | 23.433333 | 0 | 23.433333 |
| B24 | 3 | 0 | 3 | 23.433333 | 0 | 23.433333 |
| B20 | 11 | 0 | 11 | 22.589958 | 0 | 22.589958 |
| A4 | 0 | 19 | 19 | 0 | 22.154282 | 22.154282 |
| A8 | 0 | 18 | 18 | 0 | 21.839624 | 21.839624 |
| A2 | 0 | 6 | 6 | 0 | 21.812029 | 21.812029 |
| B46 | 11 | 2 | 13 | 17.744659 | 3.696833 | 21.441492 |
| D10 | 10 | 0 | 10 | 21.422572 | 0 | 21.422572 |
| B43 | 10 | 0 | 10 | 21.394686 | 0 | 21.394686 |
| A10 | 0 | 17 | 17 | 0 | 21.136032 | 21.136032 |
| B45 | 6 | 0 | 6 | 20.91645 | 0 | 20.91645 |
| A13 | 0 | 14 | 14 | 0 | 20.749829 | 20.749829 |
| B4 | 5 | 0 | 5 | 20.266667 | 0 | 20.266667 |
| C4 | 12 | 4 | 16 | 14.325138 | 5.436313 | 19.761451 |
| B28 | 2 | 0 | 2 | 17.1 | 0 | 17.1 |
| B34 | 2 | 0 | 2 | 17.1 | 0 | 17.1 |
| D7 | 5 | 0 | 5 | 16.404029 | 0 | 16.404029 |
| D5 | 2 | 0 | 2 | 15.833333 | 0 | 15.833333 |
| B16 | 4 | 0 | 4 | 15.716246 | 0 | 15.716246 |
| D3 | 6 | 1 | 7 | 13.543729 | 1.583333 | 15.127063 |
| D9 | 9 | 2 | 11 | 10.849831 | 3.8 | 14.649831 |
| A12 | 0 | 9 | 9 | 0 | 14.108363 | 14.108363 |
| D12 | 7 | 1 | 8 | 11.994127 | 1.52 | 13.514127 |
| B11 | 6 | 0 | 6 | 13.092893 | 0 | 13.092893 |
| D11 | 5 | 0 | 5 | 13.089996 | 0 | 13.089996 |
| D6 | 4 | 0 | 4 | 10.947619 | 0 | 10.947619 |
| D14 | 5 | 0 | 5 | 10.351859 | 0 | 10.351859 |
| B27 | 2 | 0 | 2 | 10.133333 | 0 | 10.133333 |
| B3 | 1 | 0 | 1 | 7.6 | 0 | 7.6 |
| B22 | 1 | 0 | 1 | 7.6 | 0 | 7.6 |
| D13 | 2 | 0 | 2 | 5.880952 | 0 | 5.880952 |
| B47 | 1 | 0 | 1 | 3.166667 | 0 | 3.166667 |
