# Supplementary material for: Comprehensive analysis method of key risk factors in the subway operation accident by complex network and accident data
Source: PLoS One. 2026 Sep 18;21(9):e0358549. doi: 10.1371/journal.pone.0358549 (PMC13588349; doi:10.1371/journal.pone.0358549)
Supplement: Supplementary file 3 [file pone.0358549.s003.docx]

**Supplementary file 3**

**Mean and standard deviation of the giant connected component (GCC) size under three attack strategies at varying node removal fractions**

| **Attack_Fraction** | **Random_GCC_mean** | **Random_GCC_std** | **MI_GCC_mean** | **MI_GCC_std** | **PR_GCC_mean** | **PR_GCC_std** |
| --- | --- | --- | --- | --- | --- | --- |
| 0 | 0.9847999999999999 | 0.006537413691803345 | 0.946666666666666 | 6.661338147750939e-16 | 0.9866666666666662 | 4.440892098500626e-16 |
| 0.013513513513513514 | 0.9706666666666673 | 0.0070553368295055985 | 0.9200000000000012 | 1.1102230246251565e-15 | 0.9733333333333342 | 7.771561172376096e-16 |
| 0.02702702702702703 | 0.9544000000000002 | 0.009648028008066935 | 0.9066666666666664 | 2.220446049250313e-16 | 0.9600000000000007 | 7.771561172376096e-16 |
| 0.04054054054054054 | 0.9402666666666658 | 0.010746368895791938 | 0.8933333333333329 | 4.440892098500626e-16 | 0.946666666666666 | 6.661338147750939e-16 |
| 0.05405405405405406 | 0.9261333333333331 | 0.011379513756454336 | 0.866666666666667 | 3.3306690738754696e-16 | 0.9333333333333325 | 8.881784197001252e-16 |
| 0.06756756756756757 | 0.9101333333333339 | 0.013547611515605922 | 0.8400000000000007 | 7.771561172376096e-16 | 0.9200000000000012 | 1.1102230246251565e-15 |
| 0.08108108108108109 | 0.8933333333333331 | 0.015549205052920774 | 0.8266666666666673 | 6.661338147750939e-16 | 0.9066666666666664 | 2.220446049250313e-16 |
| 0.0945945945945946 | 0.8775999999999999 | 0.017626243060971443 | 0.8133333333333327 | 6.661338147750939e-16 | 0.8933333333333329 | 4.440892098500626e-16 |
| 0.10810810810810811 | 0.8626666666666669 | 0.018135294011647266 | 0.7999999999999997 | 3.3306690738754696e-16 | 0.866666666666667 | 3.3306690738754696e-16 |
| 0.12162162162162163 | 0.848 | 0.01995550606279437 | 0.7733333333333333 | 0 | 0.8533333333333324 | 9.992007221626409e-16 |
| 0.13513513513513514 | 0.8335999999999999 | 0.02035070296356152 | 0.7199999999999995 | 4.440892098500626e-16 | 0.8400000000000007 | 7.771561172376096e-16 |
| 0.14864864864864866 | 0.8200000000000003 | 0.01999999999999999 | 0.4400000000000002 | 2.220446049250313e-16 | 0.8133333333333327 | 6.661338147750939e-16 |
| 0.16216216216216217 | 0.8069333333333332 | 0.019088565512718172 | 0.4400000000000002 | 2.220446049250313e-16 | 0.7999999999999997 | 3.3306690738754696e-16 |
| 0.17567567567567569 | 0.7930666666666666 | 0.01999822214320286 | 0.4266666666666662 | 4.996003610813204e-16 | 0.7600000000000002 | 2.220446049250313e-16 |
| 0.1891891891891892 | 0.7778666666666669 | 0.02006389792637514 | 0.38666666666666666 | 0 | 0.7466666666666668 | 1.1102230246251565e-16 |
| 0.20270270270270271 | 0.7637333333333332 | 0.01996263175702708 | 0.35999999999999976 | 2.220446049250313e-16 | 0.7199999999999995 | 4.440892098500626e-16 |
| 0.21621621621621623 | 0.7485333333333333 | 0.02082903955752375 | 0.3333333333333335 | 1.6653345369377348e-16 | 0.6933333333333342 | 8.881784197001252e-16 |
| 0.22972972972972974 | 0.7336 | 0.020740084645707483 | 0.2933333333333331 | 2.220446049250313e-16 | 0.6799999999999998 | 2.220446049250313e-16 |
| 0.24324324324324326 | 0.7184 | 0.019891259945569603 | 0.2799999999999998 | 2.220446049250313e-16 | 0.666666666666667 | 3.3306690738754696e-16 |
| 0.2567567567567568 | 0.7042666666666668 | 0.01990019542059266 | 0.2533333333333334 | 5.551115123125783e-17 | 0.6533333333333328 | 5.551115123125783e-16 |
| 0.2702702702702703 | 0.6896000000000001 | 0.020662795336331218 | 0.24000000000000019 | 1.942890293094024e-16 | 0.6400000000000002 | 2.220446049250313e-16 |
| 0.28378378378378377 | 0.6741333333333334 | 0.02169034603484028 | 0.24000000000000019 | 1.942890293094024e-16 | 0.626666666666666 | 6.661338147750939e-16 |
| 0.2972972972972973 | 0.6594666666666668 | 0.021698540657534255 | 0.24000000000000019 | 1.942890293094024e-16 | 0.6133333333333333 | 0 |
| 0.31081081081081086 | 0.6434666666666666 | 0.023673332394630608 | 0.19999999999999993 | 8.326672684688674e-17 | 0.6000000000000005 | 5.551115123125783e-16 |
| 0.32432432432432434 | 0.6261333333333332 | 0.024141803301880052 | 0.1866666666666667 | 2.7755575615628914e-17 | 0.5866666666666662 | 4.440892098500626e-16 |
| 0.33783783783783783 | 0.6098666666666668 | 0.0265075251370038 | 0.17333333333333356 | 2.220446049250313e-16 | 0.5599999999999996 | 4.440892098500626e-16 |
| 0.35135135135135137 | 0.5952000000000001 | 0.027937708487912088 | 0.17333333333333356 | 2.220446049250313e-16 | 0.5466666666666667 | 1.1102230246251565e-16 |
| 0.3648648648648649 | 0.5810666666666667 | 0.02958558207415676 | 0.16000000000000006 | 5.551115123125783e-17 | 0.5333333333333339 | 5.551115123125783e-16 |
| 0.3783783783783784 | 0.5656 | 0.030415200877923588 | 0.16000000000000006 | 5.551115123125783e-17 | 0.5199999999999997 | 3.3306690738754696e-16 |
| 0.3918918918918919 | 0.5509333333333334 | 0.028651468839601686 | 0.16000000000000006 | 5.551115123125783e-17 | 0.4933333333333331 | 2.220446049250313e-16 |
| 0.40540540540540543 | 0.5376000000000001 | 0.027896953238660302 | 0.16000000000000006 | 5.551115123125783e-17 | 0.48000000000000037 | 3.885780586188048e-16 |
| 0.41891891891891897 | 0.5229333333333334 | 0.028257860263414612 | 0.16000000000000006 | 5.551115123125783e-17 | 0.3733333333333334 | 5.551115123125783e-17 |
| 0.43243243243243246 | 0.5090666666666668 | 0.02880617217812105 | 0.16000000000000006 | 5.551115123125783e-17 | 0.1866666666666667 | 2.7755575615628914e-17 |
| 0.44594594594594594 | 0.4938666666666666 | 0.02871592666873985 | 0.16000000000000006 | 5.551115123125783e-17 | 0.1866666666666667 | 2.7755575615628914e-17 |
| 0.4594594594594595 | 0.4799999999999999 | 0.02822134731802231 | 0.16000000000000006 | 5.551115123125783e-17 | 0.1866666666666667 | 2.7755575615628914e-17 |
| 0.472972972972973 | 0.4653333333333333 | 0.027096535243868774 | 0.16000000000000006 | 5.551115123125783e-17 | 0.1866666666666667 | 2.7755575615628914e-17 |
| 0.4864864864864865 | 0.4522666666666666 | 0.02541600545588022 | 0.16000000000000006 | 5.551115123125783e-17 | 0.1866666666666667 | 2.7755575615628914e-17 |
| 0.5 | 0.4376 | 0.027284835022814813 | 0.16000000000000006 | 5.551115123125783e-17 | 0.1866666666666667 | 2.7755575615628914e-17 |
| 0.5135135135135136 | 0.42266666666666663 | 0.026161889160464778 | 0.14666666666666656 | 1.1102230246251565e-16 | 0.1866666666666667 | 2.7755575615628914e-17 |
| 0.5270270270270271 | 0.41013333333333335 | 0.02495293347261778 | 0.12000000000000009 | 9.71445146547012e-17 | 0.1866666666666667 | 2.7755575615628914e-17 |
| 0.5405405405405406 | 0.39733333333333326 | 0.025577768124334505 | 0.12000000000000009 | 9.71445146547012e-17 | 0.1866666666666667 | 2.7755575615628914e-17 |
| 0.5540540540540541 | 0.3818666666666667 | 0.028316936431911004 | 0.08000000000000003 | 2.7755575615628914e-17 | 0.1866666666666667 | 2.7755575615628914e-17 |
| 0.5675675675675675 | 0.368 | 0.028596814119369627 | 0.040000000000000015 | 1.3877787807814457e-17 | 0.17333333333333356 | 2.220446049250313e-16 |
| 0.5810810810810811 | 0.3514666666666667 | 0.03768972745398348 | 0.026666666666666637 | 3.122502256758253e-17 | 0.10666666666666655 | 1.249000902703301e-16 |
| 0.5945945945945946 | 0.33840000000000003 | 0.0364553913098674 | 0.026666666666666637 | 3.122502256758253e-17 | 0.10666666666666655 | 1.249000902703301e-16 |
| 0.6081081081081081 | 0.3253333333333333 | 0.03964284999284933 | 0.026666666666666637 | 3.122502256758253e-17 | 0.10666666666666655 | 1.249000902703301e-16 |
| 0.6216216216216217 | 0.31360000000000005 | 0.036780671252408886 | 0.026666666666666637 | 3.122502256758253e-17 | 0.10666666666666655 | 1.249000902703301e-16 |
| 0.6351351351351352 | 0.2992000000000001 | 0.03638412352172915 | 0.026666666666666637 | 3.122502256758253e-17 | 0.10666666666666655 | 1.249000902703301e-16 |
| 0.6486486486486487 | 0.28666666666666674 | 0.034486712417006835 | 0.013333333333333319 | 1.5612511283791264e-17 | 0.10666666666666655 | 1.249000902703301e-16 |
| 0.6621621621621622 | 0.26933333333333337 | 0.03900997023554078 | 0.013333333333333319 | 1.5612511283791264e-17 | 0.08000000000000003 | 2.7755575615628914e-17 |
| 0.6756756756756757 | 0.25626666666666675 | 0.03697831196195352 | 0.013333333333333319 | 1.5612511283791264e-17 | 0.08000000000000003 | 2.7755575615628914e-17 |
| 0.6891891891891893 | 0.24613333333333334 | 0.03437932195052395 | 0.013333333333333319 | 1.5612511283791264e-17 | 0.053333333333333274 | 6.245004513516506e-17 |
| 0.7027027027027027 | 0.23413333333333333 | 0.03157045031882399 | 0.013333333333333319 | 1.5612511283791264e-17 | 0.053333333333333274 | 6.245004513516506e-17 |
| 0.7162162162162162 | 0.22133333333333333 | 0.030051807118892382 | 0.013333333333333319 | 1.5612511283791264e-17 | 0.053333333333333274 | 6.245004513516506e-17 |
| 0.7297297297297298 | 0.2066666666666666 | 0.03479463560186637 | 0.013333333333333319 | 1.5612511283791264e-17 | 0.053333333333333274 | 6.245004513516506e-17 |
| 0.7432432432432433 | 0.19359999999999997 | 0.03764063997566697 | 0.013333333333333319 | 1.5612511283791264e-17 | 0.040000000000000015 | 1.3877787807814457e-17 |
| 0.7567567567567568 | 0.1826666666666667 | 0.03570247423732236 | 0.013333333333333319 | 1.5612511283791264e-17 | 0.026666666666666637 | 3.122502256758253e-17 |
| 0.7702702702702703 | 0.17093333333333333 | 0.0333802336720401 | 0.013333333333333319 | 1.5612511283791264e-17 | 0.026666666666666637 | 3.122502256758253e-17 |
| 0.7837837837837838 | 0.15733333333333335 | 0.03547769252173352 | 0.013333333333333319 | 1.5612511283791264e-17 | 0.026666666666666637 | 3.122502256758253e-17 |
| 0.7972972972972974 | 0.1453333333333334 | 0.03550273854851826 | 0.013333333333333319 | 1.5612511283791264e-17 | 0.026666666666666637 | 3.122502256758253e-17 |
| 0.8108108108108109 | 0.1338666666666667 | 0.03265550829832882 | 0.013333333333333319 | 1.5612511283791264e-17 | 0.026666666666666637 | 3.122502256758253e-17 |
| 0.8243243243243243 | 0.12453333333333338 | 0.029648009114347704 | 0.013333333333333319 | 1.5612511283791264e-17 | 0.013333333333333319 | 1.5612511283791264e-17 |
| 0.8378378378378379 | 0.11253333333333336 | 0.029593993384544177 | 0.013333333333333319 | 1.5612511283791264e-17 | 0.013333333333333319 | 1.5612511283791264e-17 |
| 0.8513513513513514 | 0.10320000000000004 | 0.028944084024200872 | 0.013333333333333319 | 1.5612511283791264e-17 | 0.013333333333333319 | 1.5612511283791264e-17 |
| 0.8648648648648649 | 0.09200000000000004 | 0.028503411101909275 | 0.013333333333333319 | 1.5612511283791264e-17 | 0.013333333333333319 | 1.5612511283791264e-17 |
| 0.8783783783783784 | 0.08026666666666664 | 0.026058395959843727 | 0.013333333333333319 | 1.5612511283791264e-17 | 0.013333333333333319 | 1.5612511283791264e-17 |
| 0.8918918918918919 | 0.06906666666666667 | 0.024249077874793048 | 0.013333333333333319 | 1.5612511283791264e-17 | 0.013333333333333319 | 1.5612511283791264e-17 |
| 0.9054054054054055 | 0.058666666666666686 | 0.020997354330698163 | 0.013333333333333319 | 1.5612511283791264e-17 | 0.013333333333333319 | 1.5612511283791264e-17 |
| 0.918918918918919 | 0.04933333333333335 | 0.01753726191728787 | 0.013333333333333319 | 1.5612511283791264e-17 | 0.013333333333333319 | 1.5612511283791264e-17 |
| 0.9324324324324325 | 0.03999999999999998 | 0.01436043948569201 | 0.013333333333333319 | 1.5612511283791264e-17 | 0.013333333333333319 | 1.5612511283791264e-17 |
| 0.945945945945946 | 0.029333333333333322 | 0.011623730516108464 | 0.013333333333333319 | 1.5612511283791264e-17 | 0.013333333333333319 | 1.5612511283791264e-17 |
| 0.9594594594594595 | 0.02213333333333332 | 0.009469483149101177 | 0.013333333333333319 | 1.5612511283791264e-17 | 0.013333333333333319 | 1.5612511283791264e-17 |
| 0.972972972972973 | 0.017599999999999984 | 0.006219682021168322 | 0.013333333333333319 | 1.5612511283791264e-17 | 0.013333333333333319 | 1.5612511283791264e-17 |
| 0.9864864864864865 | 0.013333333333333319 | 1.5612511283791264e-17 | 0.013333333333333319 | 1.5612511283791264e-17 | 0.013333333333333319 | 1.5612511283791264e-17 |
| 0 | 0.9847999999999999 | 0.006537413691803345 | 0.946666666666666 | 6.661338147750939e-16 | 0.9866666666666662 | 4.440892098500626e-16 |
| 0.013513513513513514 | 0.9706666666666673 | 0.0070553368295055985 | 0.9200000000000012 | 1.1102230246251565e-15 | 0.9733333333333342 | 7.771561172376096e-16 |
| 0.02702702702702703 | 0.9544000000000002 | 0.009648028008066935 | 0.9066666666666664 | 2.220446049250313e-16 | 0.9600000000000007 | 7.771561172376096e-16 |
| 1 | 0 | 0 | 0 | 0 | 0 | 0 |
